# Functional annotation of the vlinc class of non-coding RNAs using systems biology approach

**DOI:** 10.1093/nar/gkw162

**Published:** 2016-03-21

**Authors:** Georges St. Laurent, Yuri Vyatkin, Denis Antonets, Maxim Ri, Yao Qi, Olga Saik, Dmitry Shtokalo, Michiel J.L. de Hoon, Hideya Kawaji, Masayoshi Itoh, Timo Lassmann, Erik Arner, Alistair R.R. Forrest, The FANTOM consortium, Estelle Nicolas, Timothy A. McCaffrey, Piero Carninci, Yoshihide Hayashizaki, Claes Wahlestedt, Philipp Kapranov

**Affiliations:** 1 Institute of Genomics, School of Biomedical Sciences, Huaqiao University, 668 Jimei Road, Xiamen 361021, China; 2 St. Laurent Institute, 317 New Boston St., Suite 201, Woburn, MA 01801, USA; 3 Department of Molecular Biology, Cell Biology, and Biochemistry, Brown University, Providence, RI, USA; 4 AcademGene Ltd., 6, Acad. Lavrentjev ave., Novosibirsk 630090, Russia; 5 State Research Center of Virology and Biotechnology ‘Vector’, Novosibirsk, Russia; 6 A. P. Ershov Institute of Informatics Systems SB RAS, 6, Acad. Lavrentjev ave., Novosibirsk 630090, Russia; 7 Federal Research Center Institute of Cytology and Genetics SB RAS, 10, Acad. Lavrentjev ave., Novosibirsk 630090, Russia; 8 RIKEN Omics Science Center (OSC)^†^, 1–7–22 Suehiro-cho, Tsurumi-ku, Yokohama, 230–0045, Japan; 9 RIKEN Center for Life Science Technologies, Division of Genomic Technologies, 1–7–22 Suehiro-cho, Tsurumi-ku, Yokohama, Kanagawa, 230–0045, Japan; 10 RIKEN Preventive Medicine and Diagnosis Innovation Program (PMI), 2–1 Hirosawa, Wako-shi, Saitama 351–0198, Japan; 11 Telethon Kids Institute, The University of Western Australia, 100 Roberts Road, Subiaco, Subiaco, 6008, Western Australia, Australia; 12 LBCMCP, Centre de Biologie Intégrative (CBI), Université de Toulouse, CNRS, UPS, France; 13 The George Washington University Medical Center, Department of Medicine, Division of Genomic Medicine, 2300 I St. NW, Washington, DC, USA; 14 Center for Therapeutic Innovation and Department of Psychiatry and Behavioral Sciences, University of Miami Miller School of Medicine, 1501 NW 10th Ave., Miami, FL 33136, USA

## Abstract

Functionality of the non-coding transcripts encoded by the human genome is the coveted goal of the modern genomics research. While commonly relied on the classical methods of forward genetics, integration of different genomics datasets in a global Systems Biology fashion presents a more productive avenue of achieving this very complex aim. Here we report application of a Systems Biology-based approach to dissect functionality of a newly identified vast class of very long intergenic non-coding (vlinc) RNAs. Using highly quantitative FANTOM5 CAGE dataset, we show that these RNAs could be grouped into 1542 novel human genes based on analysis of insulators that we show here indeed function as genomic barrier elements. We show that vlincRNAs genes likely function in *cis* to activate nearby genes. This effect while most pronounced in closely spaced vlincRNA–gene pairs can be detected over relatively large genomic distances. Furthermore, we identified 101 vlincRNA genes likely involved in early embryogenesis based on patterns of their expression and regulation. We also found another 109 such genes potentially involved in cellular functions also happening at early stages of development such as proliferation, migration and apoptosis. Overall, we show that Systems Biology-based methods have great promise for functional annotation of non-coding RNAs.

## INTRODUCTION

Understanding pervasive transcription from the human genome and the resulting ‘dark matter’ RNA represents a fundamental challenge of contemporary biology. Due to their extensive impact in many regulatory networks, the universe of these RNAs has been called the ‘computational engine of the cell’ (1,2). We recently reported on a novel class of mammalian very large intergenic non-coding RNAs–vlincRNAs (3,4) that comprises a significant fraction of the dark matter RNA in human cells (5). These transcripts were defined from poly-A minus RNAseq or total RNAseq datasets as regions of 50 kb or more of relatively abundant intergenic transcription that have no overlap with annotated genes (3,4). These transcripts can reach ∼1 MB with a median size of ∼83.3 kb (4.8 times larger than median size of a known gene in UCSC Genes database). At least 2147 unique vlincRNAs exist, spanning over 10% of the genome. Despite being initially identified as regions of transcription and thus potentially harbouring multiple transcripts, different types of evidence suggest that vlincRNA can represent one big independently regulated transcript. First, entire transcription units have evidence of RNA presence (3,4). Second, long-range RT-PCR analysis suggests the presence of a single transcript (3). Third, RNAi-mediated inhibition of vlincRNAs can be measured large distances from the site of siRNA design (6). Finally, 5′ ends of a significant number of vlincRNAs associate with canonical RNA Pol 2 promoters suggesting that they are regulated by the same mechanisms as known genes (3). Another property of vlincRNAs is highly cell-type specific expression (4). In fact, a specific subset of vlincRNAs controlled by promoters containing sequences of endogenous retroviruses (LTRs) associates with cancers and pluripotent cells, more than conventional transcripts encoding proteins (3). This suggests a tantalizing link between these two processes via this class of ncRNAs.

While RNA depletion experiments and human–mouse synteny properties suggest functional roles for some these transcripts (3,6), the biological properties of most remain enigmatic. The sheer number and diversity of these RNAs and the even larger number of possible vlincRNA-phenotype combinations suggest the need for novel Systems Biology approaches to establish a global picture of functionality of this and other classes of ncRNAs (7). The hallmark of such approaches is integration that would take place on independent multi-dimensional genomics datasets to extract the signal and reduce both technological and biological noise (5).

We endeavoured to test this approach on the FANTOM5 Cap Analysis of Gene Expression (CAGE) dataset, a unique resource based on 5′ capture of transcripts and designed to pave the way for systems biology analysis. It combines a very large breadth of human tissues and cell lines of different origin with the highly sensitive and reproducible quantitation of single-molecule sequencing (SMS) (8,9). The accurate nature of RNA measurements on the SMS platform allows detection of relationships in the data that otherwise might go unnoticed in the context of general experimental noise (10).

In this work, we used 833 samples of the following origins: 399 normal (not of stem cell origin), 92 pluripotent (embryonic, adult, induced Pluripotent Stem, various stages of stem cell differentiation, progenitor), 332 cancerous and 10 immortalized, to show that expression profiles within the FANTOM CAGE dataset can facilitate the identification of known targets of ncRNAs. We combined this data with publicly available ChIPseq data and used a Systems Biology approach to annotate functional patterns of the vlincRNA class of ncRNAs. We show that similar to a class of activating enhancer-like RNAs (RNA-a's), many vlincRNAs likely function in part by positively regulating nearby genes in *cis* (11,12). We find that vlincRNAs represent mostly standalone transcripts regulated separately from nearby genes. In a large measure, this result stems from an analysis of the distribution of insulators—DNA elements initially discovered as genomic barriers, but whose function as such remained in question (13). We show that insulators do in fact act as genome barriers and use this result to annotate 1542 novel standalone human vlincRNA genes, of which 722 we could assign to human promoters.

Most notably, we found that 101 of vlincRNAs genes containing LTR sequences in their promoters are highly regulated by three major pluripotency-associated transcription factors (TFs)—OCT4, NANOG and SOX2. Gene Ontology (GO) analysis of all human protein-coding genes correlating with these 101 vlincRNAs revealed strong enrichment of functions associated with early embryonic development. Thus, Systems Biology analysis of independent high-throughput datasets (ChIPSeq and CAGE) reveals a consistent pattern of LTR-regulated vlincRNAs representing novel ncRNAs likely involved in early development. In addition, we revealed functional association of nonLTR vlincRNAs bound by the pluripotent factors with cellular proliferation, migration and apoptosis.

## MATERIALS AND METHODS

### Datasets

1. FANTOM5 CAGE (8): coordinates of aligned CAGE tags for this and all other analyses were taken from 1959 alignment BAM files representing 833 biological samples of the FANTOM5 dataset (http://fantom.gsc.riken.jp/5/datafiles/phase2.0/basic/).
2. UCSC Known Genes transcripts and ESTs: UCSC HG19 Annotation Database (http://hgdownload.cse.ucsc.edu/goldenPath/hg19/database/) (14). For ESTs – all (spliced and unspliced) were used, file all_est.txt.gz.
3. VlincRNAs: Supplementary Table S1 in (3).
4. LTR's: coordinates of the LTR repeats were extracted from the RepeatMasker track tables UCSC HG19 Annotation Database (http://hgdownload.cse.ucsc.edu/goldenPath/hg19/database/) (14).
5. Pluripotency TF binding sites (15): the coordinates of the ChIPseq peaks were obtained from GEO (Accession IDs: GSM1537611, GSM1537610, GSM1537612).
6. Promoters: Active, Weak and Poised promoters from 9 human cell lines (16) were downloaded from the UCSC browser (http://www.genome.ucsc.edu/cgi-bin/hgTrackUi?db=hg19&hgsid=368635859&g=wgEncodeBroadHmm).
7. Insulator elements: from the same source the dataset #6.
8. ENCODE RNAseq data (17) was downloaded from UCSC browser (http://www.genome.ucsc.edu/cgi-bin/hgFileUi?db=hg19&g=wgEncodeCshlLongRnaSeq).

### Statistical analyses and packages

Statistical analysis procedures were performed using R language and environment for statistical computing (http://www.R-project.org/). Spearman correlation, Kolmogorov–Smirnov (KS) and Mann–Whitney–Wilcoxon one-sided and two-sided tests were used as indicated.

Analysis of different genomic tracks and their intersections was performed with GenomicRanges Bioconductor package (18). GOstats (2.30.0) (19) Bioconductor package was used for GO terms enrichment analysis. AnnotationDbi (20) and GSEABase (21) Bioconductor packages were used to produce custom GO-annotation for vlincRNA genes.

### Calculation of CAGE-based digital gene expression (DGE) of vlincRNAs and exons of known genes

All the analyses below use previously reported vlincRNAs (3) constructed using RNAseq data from 14 tissues and cell lines. The vlincRNA dataset consists of 2762 vlincRNAs whose strand we could define (originating from strand-specific RNA-seq data from the ENCODE project) and 1193 vlincRNAs without strand assignment because the underlying SMS RNAseq used a not highly strand-specific cDNA synthesis protocol. Nevertheless, in this work, we defined strand of the 1193 vlincRNAs by simply comparing the number of reads aligned to either plus and minus strand of the genome in a source RNAseq library. In fact, we indeed found that DGE values based on CAGE and RNAseq tags from the sense strand of vlincRNAs always correlated slightly better than counting the tags from both strands (for example, 0.819 versus 0.804 for blood based on SMS-RNAseq). Therefore, the counts (normalized by the total number of reads and length of genomic element) of CAGE tags within the boundaries of vlincRNAs and exons of known genes on the same strand served as the corresponding DGE values throughout the entire analysis presented in this work.

We thus derived a total of 3955 (2762 + 1193) vlincRNAs. Coordinates of these vlincRNAs sometimes overlap because they were found in different tissues or cell lines (3).

Coordinates of CAGE tags were overlapped with coordinates of genomic intervals corresponding to 729 723 exons of 80 922 UCSC Genes transcripts and 3955 vlincRNAs. Intervals corresponding to UCSC Genes exons and UCSC RepeatMasker rRNA repeats were excluded from coordinates of vlincRNAs irrespective of strand on which they overlapped. This was done to exclude any signal from exons of sense short protein-coding transcripts (<5 kb) allowed to be present inside vlincRNAs by definition (3), and signal from opposite strand exons due to non-strand specific cDNA synthesis. The number of aligned CAGE tags was calculated for each exonic or vlincRNA interval in a strand specific fashion—only tags mapping to the same strand as exons or vlincRNAs were counted. A tag was assigned to an interval if the 5′ end of the former mapped inside the interval or on its border. The total number of aligned tags was summed up across different sequencing channels corresponding to the same biological sample. The number of tags was summed across exons of the same transcript to give the final number per the transcript. Since vlincRNAs and known genes of various sizes were compared to each other in the downstream analysis, the DGE CAGE counts required normalization by length of the corresponding genomic elements. This step did not change significantly the correlation between CAGE and RNAseq (data not shown). Therefore, the ‘raw DGE’ was normalized by per 1 M (genes) or 100 M (vlincRNAs) ‘informative reads’ in each sample and per 1 kb of sequence length, following the standard RPKM (reads per kilobase per million) formula

\begin{equation*} \frac{{{\rm raw\;DGE}}}{{{\rm informative\;reads \cdot length\;of\;interval}}} \cdot 10^9 ({\rm genes}) \end{equation*}

\begin{equation*} \frac{{{\rm raw\;DGE}}}{{{\rm informative\;reads \cdot length\;of\;interval}}} \cdot 10^{11} ({\rm vlincRNAs}) \end{equation*}

where ‘informative reads’ is the total number of reads in the sample uniquely aligning to the reference genome and corresponding to chr1–22, X,Y; ‘length of interval’ is the length of vlincRNA reduced by total length of overlapping UCSC Genes exons and rRNA repeats in case of vlincRNA or sum of lengths of exons in case of UCSC Genes transcripts.

### Calculation of RNASeq-based DGE for vlincRNAs and exons

The same procedure as described in the above section was used for the RNAseq data with one exception—a tag aligning across the border of an interval was counted as 0.5 tag for that interval.

### RTPCR validation of the CAGE quantitation of vlincRNAs

For this analysis, 31 vlincRNAs were randomly chosen out of 407 vlincRNA previously found in K562 (3). PCR primers were designed approximately in the middle of vlincRNA boundaries (Supplementary Table S1) using non-repetitive (as defined by RepeatMasker) regions of vlincRNAs. K562 cells were grown in RPMI-1640 medium with 10% FBS and 1% pen-strep to density of ∼1 million cells/ml. Total RNA was isolated from fresh cells using TrizolPlus system (Life Technologies) following the manufacturer's instructions. RNA concentration was assessed using Qubit 3.0 fluorimeter (Life Technologies) using Qubit RNA HS Assay Kit (Life Technologies, catalog Q32852) following the manufacturer's instructions. For DNAseI treatment, 20 μg of total RNA was mixed with 10 μl Turbo DNase Buffer (Life Technologies, catalog AM2238); 1 μl RNAseOut (Life Technologies, catalog 10777–019) and 2 μl TurboDNAse (Life Technologies, catalog AM2238) and incubated for 30 min at 37°C in total volume of 100 μl. After the DNAseI treatment, the RNA was purified using two rounds of AMPure beads (Beckman-Coulter, catalog A63880) purification with 1X volume of the beads following the manufacturer's instructions.

For first-strand cDNA synthesis, 1 μg of the DNAse-treated RNA was mixed with 80 ng of random hexamers (Life Technologies, catalog 48190–011) and 1 μl of 10 mM dNTPs in a total volume of 13 μl. The samples were denatured at 65°C for 5 min and placed directly on ice for 2 min. After that, 4 μl of 5X SuperScript III incubation buffer (Life Technologies, catalog 18080–044) and 1 μl of 0.1M DTT (Life Technologies, catalog 18080–044) were added. The samples were incubated at 15°C for 20 min. After that, the samples were moved on ice and 1 μl of RNaseOut (Life Technologies, catalog 10777–019) and 1 μl of SuperScript III (200 U/μl) (Life Technologies, catalog 18080–044) were added. The samples were moved back to 15°C and the program was skipped to the following steps: 25°C for 10 min, 40°C for 40 min, 55°C for 50 min, 85°C for 5 min and 4°C storage. Then 1 μl of RNaseH (Life Tecnhologies, catalog 18021–071) and 1 μl of RNaseIf (New England Biolabs, catalog M0243S) were added and the samples were incubated at 37°C for 30 min. The cDNA synthesis was performed in SureCycler 8800 (Agilent Technologies). The cDNA was purified using one round of AMPure beads (Beckman-Coulter, catalog A63880) purification with 1.8X volume of the beads following the manufacturer's instructions. cDNA concentration was quantified using Qubit 3.0 fluorimeter (Life Technologies) using Qubit ssDNA Assay Kit (Life Technologies, catalog Q10212) following the manufacturer's instructions.

The real-time PCR reactions were performed using 10 ng of cDNA, 5 μl PowerUp SYBR Green Master Mix (Applied Biosystems, catalog A25742), 500 nM of each forward and reverse primer (Supplementary Table S1) in 10 μl reaction volume on Agilent Technologies Stratagene Mx3005P cycler. The reaction conditions were as follows: step 1 (UDG Activation) 50°C 2 min, 1 cycle; step 2 (Dual-Lock™ DNA Polymerase Activation) 95°C 2 min, 1 cycle; step 3 95°C 15 s, 60°C 1 min, 40 cycles; step 4 (melting curve analysis) 95°C 1 min, 55°C 30 s, 95°C 30 s, 1 cycle. The specificity of amplification was confirmed by melting curve analysis for each primer pair. Each primer pair was assayed in triplicates. Analysis of the real-time data and Ct value extraction was performed using MxPro-Mx300P software v4.10 Build 389, Schema 85 using default parameters. Average Ct values were taken for further analysis. Each vlincRNA was normalized to the most abundant vlincRNA ID-1102 (Supplementary Table S1) and the normalized values were then used to calculate Spearman correlation with SMS CAGE and SMS RNAseq.

### Calculation of DGE of vlincRNAs excluding internal promoters or 5′ ends of ESTs

Genomic intervals corresponding to exons of antisense or short sense UCSC known genes and RepeatMasker rRNA repeats were excluded from vlincRNA boundaries as described above. Additionally, intervals corresponding to 5′ ends of ESTs extended by 1 kb in both directions were also excluded from vlincRNAs mapping to the same strand. Alternatively, promoters (16) extended by 1 kb in both directions were excluded from vlincRNAs overlapping them on either strand. Then RPKM values were calculated as described above considering length of vlincRNAs has been reduced by the lengths of the excluded intervals.

### CAGE tags density calculation around annotated 5′ ends of vlincRNAs

For each vlincRNA, a 10 kb interval around its 5′ end was prepared: left boundary +/−5000 nt for each of 2068 top strand vlincRNAs and right boundary +/−5000 nt for each of 1887 bottom strand vlincRNAs. Each interval was split into 20 bins of 500 bp each. The number of aligned CAGE tags was calculated for each bin of each interval on the respective strand. A tag was assigned to a bin if its 5′ end was located inside the bin. Sum across all vlincRNA intervals yielded the CAGE tags density in each bin. Before the analysis three vlincRNAs were removed as outliers with extremely high tag density: chr12:49525312–49578581:minus, chr2:43290436–43449539:minus and chr14:77402830–77490884:minus.

### Correlation between CAGE and RNASeq DGE for vlincRNAs and known genes

CAGE data for ‘chronic myelogenous leukemia cell line:K562 ENCODE’, ‘Whole blood’, ‘acute lymphoblastic leukemia (T-ALL) cell line:Jurkat’, ‘breast carcinoma cell line:MCF7’, ‘hepatocellular carcinoma cell line: HepG2 ENCODE’, ‘acute myeloid leukemia (FAB M5) cell line:THP-1’, ‘Aortic smooth muscle cell response to IL1b,00hr00min’, ‘bone marrow’, ‘Mesenchymal Stem Cells (adipose derived),00hr00min’, ‘Mesenchymal Stem Cells - bone marrow’ were taken from the FANTOM5 CAGE dataset. RNASeq SMS data was taken from (3) and (4). ENCODE RNASeq data is described above.

For CAGE and SMS RNAseq data, RPKM DGE was counted as described above. For ENCODE RNAseq data, DGE was counted on SAM files converted from BAM files with samtools. The same DGE calculation procedure as for CAGE was used, but for normalization purpose number of alignments in the SAM files mapping to chr1–22,X,Y,M was taken.

DGE values were calculated based on CAGE, SMS RNAseq and ENCODE RNAseq datasets for vlincRNAs and UCSC genes transcripts with exonic lengths of 1000 nt or longer and used to calculate Spearman correlation between expression levels in the same cell lines (Table 1).

**Table 1.** Spearman correlations between CAGE and RNAseq for UCSC Genes and vlincRNAs

	UCSC genes										VlincRNAs	
	K562	Jurkat	MCF7	HepG2	THP1	Whole blood	Bone marrow	Mesenchymal stem cell-bone marrow	Mesenchymal stem cell-adipose	AOSMC	K562	Whole blood
+/− 1000	0.774	0.871	0.836	0.880	0.831	0.882	0.776	0.800	0.777	0.830		
+/− 500	0.753	0.867	0.829	0.874	0.825	0.875	0.780	0.794	0.773	0.824		
+/− 100	0.683	0.846	0.788	0.841	0.783	0.837	0.729	0.754	0.735	0.784		
Exons	0.817	0.885	0.837	0.875	0.815	0.871	0.787	0.801	0.775	0.831		
+/− 5000											0.594	0.556
+/− 1000											0.486	0.405
+/− 500											0.434	0.393
+/− 100											0.324	0.390
VlincRNA body											0.838	0.819

### VlincRNA assignment to promoters and LTRs

A promoter (see above) from any of the three categories (‘Active’, ‘Weak’ and ‘Poised’) located within +/−5 kb from transcriptional start site of the vlincRNA was assigned to that vlincRNA using GenomicRanges Bioconductor package (18). Of the 3955 vlincRNAs, 1702 could be assigned to at least one such promoter. Promoters of 611 vlincRNAs overlapped with LTR repeats. Thus, we obtained three vlincRNA categories: ‘LTR’ (assigned to a promoter with LTR), ‘nonLTR’ (assigned to a promoter without LTR) and ‘No promoter’ (not assigned to a promoter).

### Analysis of correlation in targets of miRNAs

A set of experimentally verified miRNA targets was acquired from miRTarBase (http://mirtarbase.mbc.nctu.edu.tw/) (22) (human subset), release 4.5 from 1 November 2013. The genomic boundaries of each pre-miRNA were extended by +/−1 kb to serve as surrogate for the expression of the primary precursor of that miRNA. RPKM expression levels of all of the primary miRNAs based on +/−1 kb intervals and their targets were calculated using the FANTOM5 CAGE data on the set of 833 tissues. We then selected only miRNAs with non-zero expression in at least one of 833 FANTOM5 samples, and the same required for targets. The Spearman correlation between 369 miRNAs and 39 060 verified targets was computed based on CAGE tags in 833 FANTOM5 samples. A median Spearman correlation for each miRNA was calculated and plotted in Figure 3.

### Correlation between nearby vlincRNAs and UCSC genes

Starting with the RPKM-normalized expression of 3955 vlincRNAs in 833 FANTOM5 CAGE samples and expression of 80 922 UCSC Genes transcripts on the same dataset, we calculated a correlation matrix between these two datasets, in which columns represented vlincRNAs and rows—UCSC Genes transcripts. In the matrix, each element is a Spearman correlation between 833 expression values for a vlincRNA and a UCSC Genes transcript. In addition, a square matrix for Spearman correlations between the 80 922 UCSC Genes transcripts was calculated using custom python script based on SciPy and NumPy modules.

For each vlincRNA–gene pair, the following configurations could be defined: a gene on the same strand as the vlincRNA located upstream (‘same upstream’), nearby gene on the same strand located downstream (‘same downstream’), a gene on the opposite strand and 3′-end to the closest to 3′-end of a vlincRNA (‘opposite tail-to-tail’), a gene on the opposite strand and 5′-end to the closest to 5′-end of a vlincRNA (‘opposite head-to-head’). A nearby gene in each configuration falls into one of eight genomic distances from a neighbouring vlincRNA or a UCSC gene: 0–1 kb, 1–5 kb, 5–10 kb, 10–20 kb, 20–30 kb, 30–40 kb, 40–50 kb and more than 50 kb. For each genomic distance and each configuration, a Spearman correlation between transcript pairs (vlincRNA–UCSC gene or UCSC gene–UCSC gene) was taken from the correlation matrix, and if there were several nearby genes, median correlation for them was computed. Out of 3955 vlincRNAs only seven had nearby gene only on one side on either strand because of proximity to the border of a chromosome or chromosome arm. Also, 219 out of 80 922 genes had no nearby gene on either strand on one side. Finally, median values for each distance and configuration were calculated for each vlincRNA and UCSC gene (Table 3, Supplementary Table S4).

**Table 2.** Significance of differences in the fraction of distal LTR and nonLTR vlincRNAs compared to all informative reads

Distal LTR vlincRNAs				
	Cancer	Normal	Immort	Stem
Median fraction	0.038%	0.023%	0.059%	0.036%
	*P*-values one-sided Mann–Whitney–Wilcoxon Test			
Cancer		6.73E-14	0.1077	0.0998
Normal			0.001305	0.0008908
Immort				0.07098

Distal nonLTR vlincRNAs				
	cancer	normal	Immort	stem
Median fraction	0.045%	0.039%	0.051%	0.035%
	*P*-values one-sided Mann–Whitney–Wilcoxon Test			
Cancer		0.07288	0.1537	0.2988
Normal			0.1052	0.1725
Immort				0.05565

**Table 3.** Spearman correlations between vlincRNA–gene pairs and gene–gene pairs in different configurations and distance bins

Same upstream			Same downstream		Opposite tail–tail		Opposite head–head	
Distance	VlincRNAs–genes	Genes–genes	VlincRNAs–genes	Genes–genes	VlincRNAs–genes	Genes–genes	VlincRNAs–genes	Genes–genes
0–1 kb	0.390	0.069	0.376	0.081	0.172	0.084	0.291	0.309
1–5 kb	0.358	0.152	0.323	0.157	0.164	0.087	0.208	0.111
5–10 kb	0.353	0.149	0.241	0.151	0.188	0.098	0.148	0.114
10–20 kb	0.333	0.150	0.296	0.148	0.140	0.092	0.157	0.108
20–30 kb	0.242	0.139	0.236	0.138	0.187	0.076	0.172	0.102
30–40 kb	0.197	0.146	0.220	0.132	0.166	0.078	0.181	0.101
40– 50 kb	0.217	0.102	0.144	0.107	0.108	0.074	0.158	0.119
>50 kb	0.156	0.082	0.135	0.085	0.147	0.067	0.130	0.086
All^a^	0.200	0.107	0.189	0.109	0.150	0.076	0.146	0.110
*P*-values^b^		1.80E-89		3.01E-50		3.63E-63		3.88E-03

^a^Correlations calculated by combining all transcript pairs.

^b^One-sided Mann–Whitney–Wilcoxon test for difference in correlation distributions between all vlincRNA–genes and gene–gene pairs within the configuration.

### Insulator analysis

A joined set of genomic insulators was produced from insulator elements found in nine cell lines (see above). Two transcripts (vlincRNAs or UCSC genes) were considered separated by insulator, if any genomic insulator from the set occurred between them according to their hg19 coordinates and considered not separated otherwise. Pairs of nearby transcripts defined above were split into two sets: nearby genes separated by insulators and nearby genes not separated. For these two sets, median Spearman correlations were calculated for each genomic distance and configuration for genes and vlincRNAs (Supplementary Table S4, Table 4).

**Table 4.** Difference in Spearman correlations between vlincRNA–gene pairs and gene–gene pairs separated by insulators^a^

Distance	Same upstream	Same Downstream	Opposite tail-to-tail	Opposite head-to-head
0–1 kb	0.3295	0.304	0.121	0.4535
1–5kb	0.19225	0.184	0.0925	0.1635
5–10 kb	0.1575	0.044	0.0675	-0.016
10–20 kb	0.13925	0.128	0.014	0.0395
20–30 kb	0.079	0.08575	0.0935	0.063
30–40 kb	0.0395	0.097	0.071	0.05825
40–50 kb	0.0895	0.00075	0.028	0.012
>50 kb	0.067	0.042	0.076	0.035
*P*-values^b^	5.80E-61	2.01E-35	5.51E-91	6.15E-29

^a^The numbers are Spearman correlation of vlincRNA–gene pairs minus Spearman correlation of gene–gene pairs, both types of pairs contain insulators.

^b^One-sided KS test for difference in correlation distributions between vlincRNA–genes and gene–gene pairs separated by insulators.

### Building vlincRNA genes

A vlincRNA was designated as a standalone transcript if it did not have a UCSC Gene not separated by an insulator element within 50 kb from either 5′ or 3′ direction. Overall, for 1753 vlincRNAs the nearest gene was over 50 kb away from 5′ and 3′ ends; 81 vlincRNAs were separated from the nearest gene by insulators on both 5′ and 3′ ends, and 607 vlincRNAs had a gene over 50 kb away on one end and an insulator element on another. Therefore, out of 3955, 2441 vlincRNAs were designated as standalone transcripts. The coordinates of 2441 vlincRNAs were then merged in a strand-specific fashion to yield 1542 standalone vlincRNA genes with unique coordinates (Supplementary Table S6).

### Analysis of interval overlap between different genomic datasets

The genomic overlap analysis was performed with Bedtools suite version 2.22.1 using ‘overlap’ function on sorted BED files containing coordinates and genomic strand information of all regions in two datasets to be compared. The same suite was then used to calculate the *P*-values of overlapping events based on Fisher's exact test (‘fisher’ function). We used this procedure to overlap ENCODE promoters of 1542 vlincRNA genes with 3043 ‘non-annotated stem transcripts’ (NASTs) from Fort *et al*. (23) or vlincRNA genes with 60 mouse macroRNAs (24) mapped onto hg19 coordinates. The method relies on distribution of the intervals throughout the entire sequenced portion of the genome. This assumption is applicable for NASTs, many of which mapped to introns of known genes, and mouse macroRNAs. However, most of vlincRNA genes are located in intergenic space defined in a strand-specific fashion and thus could overlap known genes on opposite strand. To address this issue, we recalculated theoretical *P*-value under assumption of uniform distribution of vlincRNA genes in intergenic space with a method developed earlier and tested with randomly generated intervals (3). Three out of 1542 vlincRNA genes were found to overlap the mouse macroRNAs in the genic space. In addition, theoretically 3.62 vlincRNA genes are expected to overlap randomly distributed macroRNAs intervals in the intergenic space. To get upper-bound *P*-value estimation, we used binomial distribution with 1542 trials, 15 successes (the actual number of vlincRNA genes overlapping the macroRNAs) and probability 0.0043 ((3.62+3)/1542) of success in each trial. Binomial *P*-value equals to 0.0034 which is in general consistent with Fisher's exact test *P*-value of 0.004 given by Bedtools.

### Transcription factor analysis

Significance of ChIPseq tag count differences between vlincRNA +/− 5 kb flanking regions and random genomic intervals was assessed with simulation tests against 1542 random non-overlapping 10 kb regions in hg19 genome (1000 permutations).

Promoters assigned to vlincRNAs genes (located with +/−5 kb from 5′ end) or UCSC Genes transcripts (located with +/−1 kb from 5′ end) were intersected with OCT4/POU5F1, SOX2, NANOG binding sites to give ChIPseq+ or ChIPseq- status to the corresponding vlincRNA or UCSC Gene transcripts. Overall, 28 479 UCSC Gene transcripts could be associated with 20 255 promoters. Among them 1419 promoters were associated with SOX2 transcription factor binding sites (TFBSs), 3961 with OCT4, 3426 with NANOG and 795 promoters had all three TFBSs. And, for 722 vlincRNA genes associated with promoters, 98 have SOX2 binding sites, 138 have OCT4, 153 have NANOG and 65 had all three TFBSs (Supplementary Table S6).

Spearman correlation coefficients between expression values of UCSC Genes, vlincRNAs and the three TFs of interest: SOX2 (uc003fkx.3), OCT4 (uc003nsv.3) and NANOG (uc009zfy.1) were calculated in two human embryonic stem (ES) cells (HES3 and H9) differentiation time-courses (9).

### GO analysis of UCSC genes correlating with vlincRNAs

For each of the 1542 vlincRNA genes, we obtained a list of GO terms enriched in UCSC Genes either correlating (Spearman correlation > = 0.35) or anti-correlating (Spearman correlation < = −0.35) with that vlincRNA gene using GOstats (2.30.0) (19) Bioconductor package (Supplementary Figure S7). Only GO terms enriched with unadjusted *P*-value threshold of <0.05 were recorded as associated with a particular vlincRNA gene. Correlating and anti-correlating GO terms were recorded separately. Using Bioconductor packages AnnotationDbi and GSEABase (see above), these correlation tables were used to produce custom GO-annotation subsequently used with GOstats for GO terms enrichment analysis as described in GOstats manual (http://www.bioconductor.org/packages/release/bioc/vignettes/GOstats/inst/doc/GOstatsForUnsupportedOrganisms.pdf). To obtain GO-enrichment analysis of different vlincRNA subsets (for example, LTR ChIPseq+), we used with GOstats and GO terms associated with all 1542 annotated vlincRNAs as a background. Only biological process GO terms were considered. False Discovery Rate (FDR)-correction was used to adjust the final *P*-values reported in Supplementary Table S8. Description of some columns in this table: ‘Padj’ = *P*-value adjusted for multiple testing; ‘ExpCount’ = number of upregulated (or downregulated) vlincRNAs with this GO term expected by chance; ‘Count’ = observed number of upregulated (or downregulated) vlincRNAs with this GO term; ‘Size’ = total number of vlincRNAs with this GO term.

### VlincRNA subcellular localization analysis

Exponentially growing K562 cells were collected and fixed following the protocol recommended by Advanced Cell Diagnostics (ACD, Hayward, USA) for RNAscope (25) assay of Non-Adherent Cells. Cells were then cyto-centrifuged onto slides using Thermo Scientific™ Shandon™ EZ Megafunnel™. Slides were air-dried for 20 min, immersed in 50% ethanol for 5 min, in 70% ethanol for 5 min, in 100% ethanol for 5 min, stored in fresh 100% −20°C ethanol and shipped to ACD. Cell staining was performed by ACD using 20 double Z target probe pairs hybridizing to a 1 Kb region of the target RNA. The hg19 coordinates of the regions chosen for probes were chr8:91319651–91320841 and chr5:53627148–53628102 for vlincRNA genes #898 and #1501 correspondingly (Supplementary Table S6). The cells were counterstained with DAPI to image the chromatin, and then coverslipped with a photoprotective solution. The cells were visualized on a Zeiss LSM 510 confocal microscope using typically 18 slices in the z dimensions through the cell. FISH probes were imaged with excitation and emission filters optimized for Atto500 (red) and then reimaged for DAPI (blue). The FISH and DAP images were merged, and z-stacks flattened as needed in Velocity software. The images shown were imaged at identical power and gain settings.

## RESULTS

### Measuring vlincRNA expression using CAGE

**Figure 1.**
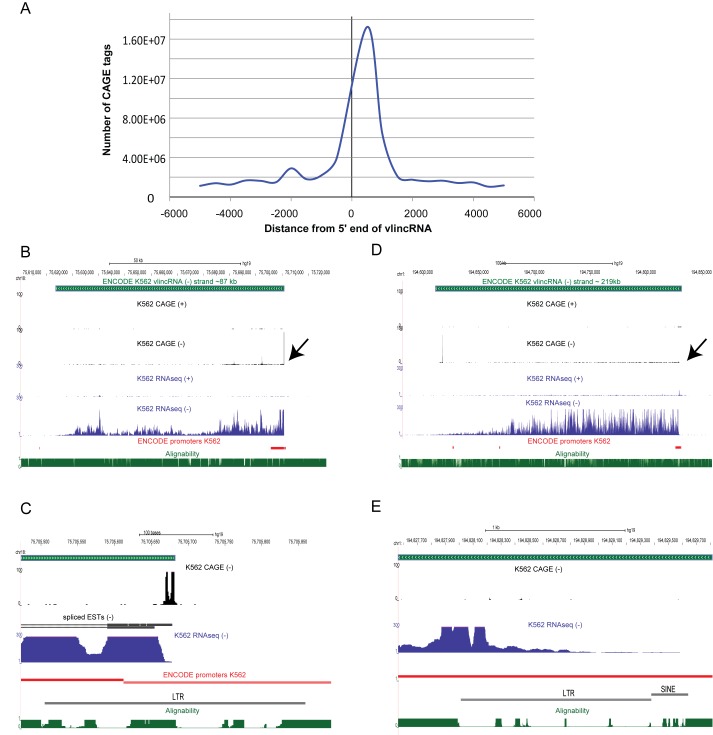
Detection of vlincRNAs using CAGE. (**A**) Cumulative plot of CAGE tags from all 833 samples around the annotated borders of 3955 individual vlincRNAs, where 3955 is a sum of 2762 strand specific vlincRNAs and 1193 vlincRNAs with calculated strand. Coordinates of vlincRNAs are from (3). The position ‘0’ (X-axis) indicates 5′ end of a vlicnRNAs, while the negative values represent base pairs in the 5′ flanking regions. For each base pair, CAGE tags were summed up across all vlincRNAs and all samples (Y-axis). Three outlier vlincs (chr12:49525312–49578581: minus, chr2:43290436–43449539: minus, chr14:77402830–77490884: minus) with extremely high CAGE expression were not used. The CAGE method can successfully detect the 5' end of vlincRNA ID-1102 (**B,C**) but not vlincRNA ID-1209 (**D,E**). Both vlincRNAs were initially found in K562 cell line ((3), Supplementary Table S1). ENCODE nuclear polyA- RNAseq track and CAGE data both from K562 cell line are shown. ENCODE alignability track for 36 mers is also shown. The ENCODE/Broad promoters represent combined promoters from ‘Active’, ‘Weak’ and ‘Poised’ categories (16).

The entire analysis presented here depends on accurate measurement of levels of protein-coding mRNAs and especially non-coding vlincRNAs, since no one has ever attempted measuring such long transcripts using CAGE. The FANTOM5 CAGE data is based on obtaining short sequence tags from 5′ ends of transcripts containing 5′ cap using Helicos SMS platform. The resulting 5′ tags are mapped back to the genome and thus represent 5′ ends of coding and non-coding RNAs if the latter contain 5′ cap. Overall, the 5′ ends of vlincRNAs showed a pronounced association with CAGE tags, as shown by the cumulative distribution of all tags around all 5′ ends of vlincRNAs (Figure 1A, also Figure 1B and C for a specific example). On the other hand, the example in Figure 1D and E highlights a problem in the detection of vlincRNAs: reliance only on CAGE tags mapping around the 5′ end of a transcript that falls within a repetitive region with a poor alignability score—for example, an LTR. In fact, a significant fraction of vlincRNAs associate with LTR containing promoters (3), thus presenting a problem for accurately quantifying these transcripts with tags mapping only near 5′ ends. The presence of repeats that frequently drive expression of other types of ncRNAs (23,26,27) makes this a more global problem. Therefore, we explored various CAGE tag counting strategies for vlincRNAs and for mRNAs, by comparing them with SMS RNAseq data in selected samples. To our knowledge, this work provides the first direct comparison of CAGE versus RNAseq both performed on the SMS platform (28).

CAGE counts for different transcripts were obtained in two different ways: (i) ‘5′ flanking’—summing up tags located within varying distances from 5′ ends—the classical CAGE approach (8)—or (ii) ‘internal’—the standard RNAseq analysis where only tags mapping inside transcripts (exons of UCSC Genes or vlincRNA boundaries) were counted. The internal method relied on the known phenomenon of post-transcriptional capping of cleaved RNAs (29–32) thus resulting in CAGE tags in the bodies of transcripts and not only at their initiation sites. To test whether this is true for vlincRNAs, we split sequence of each vlincRNA and flanking genomic regions into bins normalized by length and plotted aggregate distributions of CAGE and RNAseq reads in all K562 vlincRNAs for each bin (Supplementary Figure S1). The CAGE signal while enriched at the 5′ ends of vlincRNAs also covered fairly well the bodies of vlincRNAs and dropped in the flanking genomic regions (Supplementary Figure S1). In fact, the CAGE and RNAseq profiles looked similar—higher at the 5′ ends and gradually decreasing towards the 3′ ends—and the Spearman correlation between the CAGE and RNAseq distributions was indeed quite high: 0.947 (Supplementary Figure S1). If the CAGE signal represented only the sites of transcription initiation, the correlation with RNAseq would be low. Instead, this result argues that the internal CAGE tags predominantly represent sites of internal cleavage and capping rather than internal initiation. This observation encouraged us to compare whether the quantitation using internal CAGE tag counting method would give similar results with the RNAseq method.

To compare CAGE versus RNAseq in mRNA detection, for each known UCSC Genes transcript of 1000 nt or longer, we calculated four DGE CAGE values for +/–1 kb, +/–500 bp and +/–100 bp around annotated 5′ ends—the ‘5′ flanking’ counting method—as well as by summing up CAGE tags in all annotated exons—the ‘internal’ method. Noteworthy, integrating the tags around +/−500 bp has been the preferred method of estimating gene expression so far using for CAGE-based data (8). These values were then compared to DGE values based on RNAseq counts calculated by summing up RNAseq reads mapping to the sense strand of annotated exons. Table 1 shows the corresponding Spearman correlations. Notably, for most samples, the method of counting did not make substantial difference even though it was consistently higher in the ‘internal’ mode. However, for human leukemia cell line K562, the ‘internal’ method gave an appreciably higher correlation with RNAseq. For example, the corresponding values for +/−500 bp ‘5′ flanking’ and ‘internal’ were 0.753 and 0.818. Supplementary Figure S2 shows the scatter plots of internal CAGE versus RNAseq counting methods.

We also calculated CAGE DGE counts for vlincRNAs initially found in K562 and whole blood (3) using the two methods of counting: around the predicted 5′ ends and inside vlincRNA borders. Since our knowledge of vlincRNA 5′ end is less precise than for known genes, we used a +/−5 kb clamp around the 5′ ends to estimate the CAGE DGE counts. The comparison was done to RNAseq counts obtained by summing up reads within vlincRNA bounds. The Spearman correlations with RNAseq were much higher for the ‘internal’ method of counting (Table 1). Using window sizes similar to the ones used for known genes in the ‘5′ flanking’ method decreased the correlations even further (Table 1). Overall, for both tissues, the correlations between CAGE and RNAseq in vlincRNAs counted within the vlincRNA boundaries were >0.8 (Table 1), which made us confident that CAGE offers a reliable estimate of expression for this novel class of very long non-coding RNAs. The lower CAGE versus RNAseq correlation found in blood vlincRNAs (0.819 versus 0.838 in K562, Table 1) could be explained by their lower expression levels compared to K562 vlincRNAs (average expression of 0.208 versus 0.34, Mann–Whitney–Wilcoxon test *P*-value = 1.29 × 10^−10^).

In addition, the ‘internal’ method was also significantly more sensitive in vlincRNA detection (Supplementary Figure S3). In five cell types, the internal method has consistently detected more vlincRNAs with higher counts. This could be explained by one limitation of CAGE—the peak of the signal is limited to a very specific region of the corresponding transcript and if such region has properties that interfere with detection of reads—for example, sequence properties that interfere with sequencing or mapping of reads—then no signal is obtained. Such problem is illustrated in Figure 1D and E, where 5′ end of a vlincRNA falls within a repetitive region with a poor alignability score. The ‘internal’ counting method alleviates this problem.

Given the critical importance of accurate measurements for all downstream work, we did additional tests of robustness of the internal CAGE counting method. One can imagine that presence of overlapping transcripts could complicate accurate quantitation. Therefore, first, we asked a question whether internal initiation sites would bias vlincRNA quantitation. To address this, we removed CAGE tags and RNAseq reads that fell within +/−1 kb of (i) promoters and (ii) 5′ ends of ESTs annotated within boundaries of vlincRNAs and recalculated the vlincRNA expression levels using the internal method. For this and all other analyses in this paper, we will be using the promoter list derived from the chromatin state segmentation analysis (16,33). In neither case was the correlation between CAGE and RNAseq significantly affected. In whole blood, the correlation changed from 0.819 to 0.821 in (i) and to 0.809 in (ii). And, in K562 it changed from 0.838 to 0.836 in (i) and to 0.854 in (ii). Second, we asked a question whether the presence of multiple known transcript isoforms can alter the correlation for known genes. In fact, the lowest correlation of 0.848 (compared to 0.871 for all genes) was observed in blood RNA for UCSC genes with one isoform, likely because of low level expression of such genes (data not shown). CAGE versus RNAseq correlation for genes with more than two and five isoforms was 0.865 and 0.856, respectively. Overall, these results suggest that internal initiation or presence of multiple isoform does not skew our CAGE analysis.

To further validate our counting method, we compared CAGE data with ENCODE RNAseq data in their quantification of vlincRNAs. In addition to the difference in methods of RNA analysis (CAGE versus RNAseq), the two datasets were obtained on different batches of cells, different sequencing platforms (SMS and Illumina) and in some cases on different RNA sub-fractions. To account for these differences, we have limited our analysis to cell lines for which we had SMS RNAseq data so that we could establish maximum possible correlation one could achieve if the comparison was limited to RNAseq method. Thus, we have first compared SMS RNAseq for whole cell total RNAs for K562 and HepG2 with ENCODE RNAseq for K562 (whole cell total RNA) and HepG2 (whole cell long polyA- RNA). We then estimated the same correlations between CAGE (using the internal method) and ENCODE RNAseq and asked a question how close SMS CAGE was to SMS RNAseq in correlating with ENCODE RNAseq samples. The correlations for K562 were 0.829 (SMS RNAseq versus ENCODE RNAseq) and 0.772 (SMS CAGE versus ENCODE RNAseq). The corresponding correlations for HepG2 were 0.746 and 0.7. While there was a drop in correlation due to the change in the method of analysis, this result suggests that SMS CAGE is quite close to SMS RNAseq in its correlation with the ENCODE data for vlincRNAs.

Finally, we validated the results of internal CAGE counting method using real-time RTPCR (Supplementary Table S1). We have estimated relative expression levels of 31 vlincRNAs in K562 RNA using real-time RTPCR and compared these values with internal CAGE and SMS RNAseq counting methods (Supplementary Table S1). The corresponding Spearman correlations were 0.802 (RTPCR versus CAGE) and 0.847 (RTPCR versus RNAseq).

All the results above support the notion that CAGE could be used for accurate quantitation of known genes and vlincRNAs. Because of the far superior correlations and better sensitivity for vlincRNAs, we have also adopted the ‘internal’ counting mode for the downstream analysis in this study.

### Association of vlincRNAs regulated by retroviral promoters with cancer and pluripotency

The availability of the much broader FANTOM5 CAGE dataset (833 different samples) allowed us to comprehensively establish the association of vlincRNAs with LTRs in their promoters with malignancies and pluripotency (3). We have previously found that vlincRNAs tend to be highly cell-type specific (3,4), therefore we suspected that a metric that relies on median or average would not be informative for this analysis. Indeed, distributions of tumour/normal ratios based on median or average expression of all vlincRNAs among 399 normal (excluding stem cells) or 332 cancer samples did not reveal any differences between LTR and nonLTR vlincRNAs (Supplementary Figure S4). The same effect was obtained when using 95th or 98th percentiles of expression (data not shown). Only comparison of distributions of ratios based on maximum expression values (maximum in tumours versus maximum in normal tissues) for each vlincRNA could separate normal and cancer tissues (Supplementary Figure S4, KS test *P*-value <2.2 × 10^−16^). The distribution of maximums in the cancer tissues was consistently higher only for LTR vlincRNAs (Supplementary Figure S4). Therefore, we calculated a ratio of maximum expression in cancer versus maximum in normal tissues (RMECN) for each of 1702 out of 3955 vlincRNAs that could be assigned to promoters (Supplementary Table S2). The median RMECN value was 1.706 for vlincRNAs with LTRs (‘LTR’) and 1.132 for those without LTRs in the promoters (‘nonLTR’) (KS test *P*-value = 1.308 × 10^−10^, Mann–Whitney–Wilcoxon test *P*-value = 8.473 × 10^−11^) showing the more significant upregulation of LTR vlincRNAs in cancerous state as compared to the nonLTR vlincRNAs. The main reason for the RMECN difference between the LTR and nonLTR vlincRNAs is the lower expression of the former in the normal tissues rather than higher expression in cancers. The average of maximum expression values for LTR and nonLTR vlincRNAs in cancers were respectively 110.5 and 106.1 and in normal tissues 62.7 and 104.0. This suggests that LTR-driven vlincRNAs are silenced in normal cells and then activated in specific cancers (and pluripotent stem cells—see below), while nonLTR vlincRNAs can be highly expressed in either tissue type.

A maximum-based metric could potentially be biased by a few outliers. Therefore, we asked a question in how many cancer cell lines would at least one LTR-based vlincRNA reach its expression maximum if all cancerous and normal samples are compared together. In fact, this happens in 106 out of 332 cancer cell lines (31.9%) compared to 68/399 in normal samples (17%). Therefore, the RMECN signal is based on 106 samples arguing against spurious effects of few cancer cell lines. We next tested this by permuting labels of cancer and normal samples and calculating RMECN for LTR and nonLTR vlincRNAs and then taking the ratio of former versus the latter. In each of 1 million permutations, the ratio of RMECN for LTR vlincRNAs to that of nonLTR vlincRNAs was lower than in the real data. We next removed 10 cancer cell lines with the highest number of extremely expressed LTR vlincRNAs and repeated the permutation analysis. The result was the same (*P*-value of 10^−6^).

On the other hand, the higher RMECN value for LTR vlincRNAs could still be explained by relatively few vlincRNAs with very high maximum values. Therefore, we have then asked whether the fraction of LTR vlincRNAs with maximum expression in a cancer sample is higher than such fraction of nonLTR vlincRNAs. Indeed, 406/611 (66.4%) LTR vlincRNAs have maximum expression in a cancer compared to 614/1091 (56.3%) nonLTR vlincRNAs (*P*-value = 2.3 × 10^−5^, Fisher's exact test). The difference becomes even larger if we remove vlincRNAs that reach maximum expression in either stem or immortalized cell lines with the corresponding values for LTR and nonLTR vlincRNAs of 393/588 (66.8%) versus 589/1047 (56.3%) (*P*-value = 1.6 × 10^−5^, Fisher's exact test). All these results support the notion that the observed upregulation of LTR versus nonLTR vlincRNAs based on RMECN are not due to a few outlier samples or vlincRNAs.

Second, we used global metrics based on the relative mass of these ncRNAs. These metrics would assume behaviour of all LTR-driven vlincRNAs as one family in each sample and we tested how well these metrics perform to separate various types of biological samples. To make sure that promoters assigned to vlincRNAs could not associate with known genes, we focused on 1077 (out of 3995) vlincRNAs separated from known genes by at least 50 kb—we will refer to them as distal vlincRNAs. We calculated the normalized fractions of CAGE tags falling into either LTR or nonLTR class, and used them to sort the 833 samples (Supplementary Table S3). Of the top 100 tissues ranked by their distal LTR-associated vlincRNA fraction, 86 (out of 332) were cancerous, 12 (out of 92) were either pluripotent or points of stem cell differentiation time courses and only 2 (out of 399) were normal (expected 40, *P*-value 3.9 × 10^−24^, Fisher's exact test). On the other hand, the same analysis based on distal nonLTR-associated vlincRNA fraction did not enrich for cancers: of the top 100 tissues, 44 samples were cancers, 10 were of stem cell origin and 46 were normal (the corresponding *P*-value of cancer sample enrichment was 0.2133).

Third, we then calculated a different summary metric—the fraction of CAGE tags falling into these two classes—distal LTR- and nonLTR-associated vlincRNAs—relative to all (not just vlincRNA) informative (uniquely mapping nuclear non-rRNA) CAGE tags for each tissue (Supplementary Table S3). The corresponding median values for normal, cancerous and stem (any kind) tissues were 0.023%, 0.038% and 0.036% for the LTR-driven vlincRNAs (see distributions of these values in Supplementary Figure S5). Applying the Mann–Whitney–Wilcoxon test to this metric also showed that LTR vlincRNAs were indeed significantly enriched in cancers and stem cells relative to normal samples (Table 2). Interestingly, despite the small sample number, the 10 immortalized cell lines also had significantly higher fraction of LTR vlincRNAs than normal samples (Table 2). In contrast, the fraction of nonLTR vlincRNAs did not distinguish the different types of tissues, underscoring a more constitutive presence of this subgroup of vlincRNAs (Table 2).

**Figure 2.**
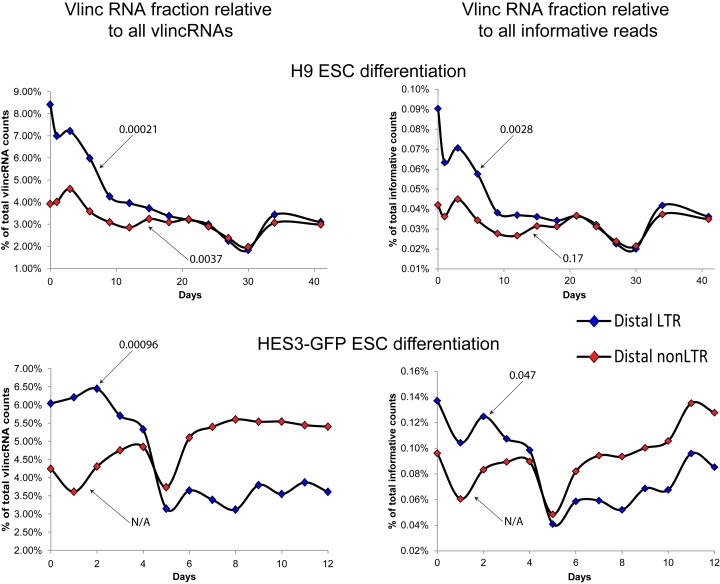
Distribution of fractions of distal LTR and nonLTR vlincRNAs during time course of embryonic stem cell differentiation. The fractions of distal (>50 kb) LTR (blue diamonds) and nonLTR (red diamonds) vlincRNAs at every time point were calculated as the ratio of number of reads relative to all informative reads (right) or all 3955 vlincRNA reads (left). Number of reads for vlincs were calculated without exon and rRNA in vlincs. The fractions (Y-axes) were plotted as functions of time of differentiation (X-axes) of two human ESC lines H1 (top) and HES-GFP (bottom). The numbers indicate *P*-values for the decreasing trend using Fisher F-test. N/A—the actual trend was to increase.

Finally, the availability of two ES differentiation time courses (9) allowed for a more direct proof of association between LTR vlincRNAs and pluripotency. The fractions of distal LTR-driven vlincRNAs decreased as H9 Embryonic Stem Cell (ESC) differentiation progressed. They also dropped during HES3 differentiation (Figure 2). A similar decrease was not observed for the fraction of distal nonLTR vlincRNAs (Figure 2). These results show a progressive loss of expression of LTR-driven vlincRNAs as measured by their relative mass during the differentiation of ESCs and serve as additional evidence of their function during pluripotency. Altogether those results establish the association vlincRNA with LTRs in their promoter with cancer and pluripotency based on 833 human samples.

### CAGE analysis can measure correlation between non-coding RNAs and their targets

**Figure 3.**
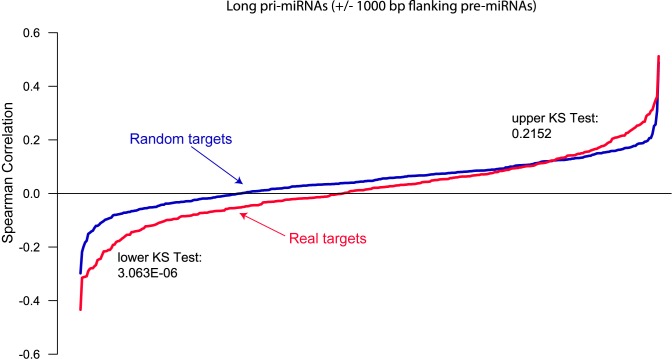
Distribution of Spearman correlations between experimentally validated and random targets of miRNAs. For each miRNA in miRTarBase, median Spearman correlations between its experimentally validated (red) and random (blue) targets were plotted for the primary forms of miRNAs. The *P*-values from the KS test that the distribution of the correlations for the real targets is either lower or higher than that for random targets are shown.

Many ncRNAs act by regulating other RNAs either directly or indirectly. Although evidence of this regulation should exist in the FANTOM5 dataset, relatively few long non-coding RNAs have a reliable list of associated targets, especially those of a novel class such as vlincRNAs. miRNAs on the other hand offer perhaps the best characterized list of targets of any class of ncRNA. Therefore, we wanted to test whether analysis of the FANTOM dataset could detect the effect of ncRNAs on their targets, using miRNAs and their predicted targets as a positive control. Therefore, we tested for correlation between the levels of the long primary precursors of miRNAs and experimentally validated mRNA targets of corresponding miRNAs (22). We counted CAGE tags in +/−1 kb window around a pre-miRNA (on the sense strand) as estimate of the corresponding pri-miRNAs. The levels of the mRNAs were calculated by counting CAGE tags within the corresponding exons. We then selected a subset of primary miRNAs expressed in at least 1 of 833 samples of the FANTOM5 dataset. Spearman correlation between each of the 369 miRNAs and each of its targets was then calculated in the 833 tissues, and compared to a control analysis conducted with random targets. Only targets expressed in at least 1 of 833 samples were taken. The plots of median correlations for the real and random targets revealed an interesting picture. As expected, the correlations for the real targets had a strong negative tendency, significantly more displaced towards lower territory than the random targets (*P*-value = 3.1 × 10^−6^, one-sided KS-test) (Figure 3). Similar conclusions were observed by the analysis of the expression levels of the mature forms of miRNAs (data not shown).

Thus, the data above suggests that the FANTOM5 dataset contains information on functional connections between long or short ncRNAs and their targets. While this conclusion was achieved based on a class of ncRNAs (miRNAs and their precursors) with a highly characterized mechanism of action and set of targets, it encouraged us to explore potential targets of vlincRNAs, a much less characterized and understood class of ncRNAs, using similar correlation-based approaches as described in the sections below.

### Patterns of correlation of vlincRNAs with adjacent genes

Although vlincRNAs proximal to known genes could be involved in important biological functions, they could also represent un-annotated introns and exons of yet undiscovered extended isoforms of known genes (34,35). Therefore, we explored the *cis* correlation between vlincRNAs and nearest genes as a function of configuration and distance (Table 3). Four possible configurations were tested: same strand vlincRNA upstream or downstream of the nearest gene and opposite strand head-to-head or tail-to-tail configurations. Numbers of vlincRNAs and genes in each configuration are given in Supplementary Table S4. Eight different distance bins were used, ranging from 0–1 kb representing adjacent vlincRNAs and genes to >50 kb, where vlincRNAs likely represent standalone genes. As a control, we used correlations between known genes in the same genomic configuration and distance (Table 3).

The first observation concerned a relatively high Spearman correlation between vlincRNAs and known genes on the same strand. The adjacent vlincRNA and genes (distance of 0–1 kb, Table 3) had the highest correlation, which decreased as distance increased (Table 3). The correlation peaked at 0.38–0.39, making it similar to the global correlation between exons of annotated genes and their corresponding introns (0.4) (36). By comparison, nearby annotated genes on the same strand did not show this pattern: their correlation ranged from 0.07 to 0.16 and changed very little with distance (Table 3), as would be expected for independently regulated units. Therefore, some vlincRNAs on the same strand and close to known genes likely represent yet un-annotated extensions of the latter (see below).

However, examination of the Spearman correlations between transcripts on opposite strands—excluding the fact that vlincRNAs could be un-annotated extensions—revealed an un-expected observation: a consistently higher correlation between nearby vlincRNA–gene pairs relative to gene–gene pairs of the same relative position and distance (Table 3). The correlations between tail-to-tail and head-to-head vlincRNAs and gene pairs across all distance bins were 0.15 and 0.146. The same numbers for the gene-to-gene pairs were 0.076 and 0.110 (Table 3). The higher positive correlations for vlincRNA–gene pairs were also statistically more significant than gene–gene pairs in all configurations (Table 3).

While shared regulatory sequences in a bidirectional promoter could potentially explain the correlation of the head-to-head orientation, this trivial explanation cannot explain the higher tail-to-tail correlation. Rather, it suggests different mechanisms by which vlincRNA affects expression of nearby genes in *cis*, a mechanism previously suggested for another class of ncRNAs—activating RNAs (11,12). It also suggested that some of the correlation between vlincRNAs and genes on the same strand could potentially be due to that mechanism rather than being trivially explained by vlincRNAs representing novel extensions of the adjacent genes. To our knowledge this represents the first analysis of the global effect of a class of ncRNAs on their neighbouring genes. To explore this in more detail, we took advantage of insulators—genetic elements known to function at least in part as barriers between different chromatin domains (13).

### Insulators support functionality of vlincRNAs as activators of adjacent genes in *cis*

Before investigating behaviour of vlincRNAs and genes separated by insulators, we wanted to ensure that the CAGE dataset provided evidence for function of insulators as genomic barriers. In fact, recent data questioned the role of insulators as barriers between genes (13). We took advantage of the insulator elements identified based on chromatin state segmentation of ChipSeq data by Hidden Markov Model (ChromHMM) in nine cell lines (16,33). Spearman correlations between pairs of vlincRNAs and genes in different genomic configurations separated and non-separated by insulator elements were calculated essentially as above. The same was done for pairs of known genes (Supplementary Table S4). The presence of an insulator element had a strikingly different effect on correlations between different types of nearby elements. As expected from a barrier element, insulators significantly reduced the correlation between adjacent head-to-head pairs of genes from 0.315 to 0.0415 (Supplementary Table S4). It is worth noting that while correlation of gene expression was calculated among all 833 samples, the insulator elements were limited to nine cell lines in which they were originally identified. Still, the observed effect suggests that an insulator functions in more than one cell line. Considering the high probability of co-regulation of two adjacent head-to-head genes by shared promoter elements, the ability of this type of element to nullify the co-regulation proves that it functions as a genomic barrier. This property makes this element quite useful in trying to separate expression correlations based on chromatin accessibility and/or promiscuous transcription from true biologically meaningful relationships.

Insulators had little effect on all other configurations of known genes, including nearby gene–gene pairs on the same strand (Supplementary Table S4). While surprising at first, the reason for this un-expected behaviour became apparent when we explored expression levels of genes not separated by insulators. In all configurations, expression of UCSC Genes not separated by insulators was significantly lower than pairs separated by the elements. This was indicated by a much larger fraction of the UCSC Genes pairs where both members were not expressed in any of the 833 tissues (Supplementary Table S5). Thus, presence of insulators correlates with expression of the loci they separate. Overall, this general characteristic caused the correlations between pairs of genes, not separated by an insulator, to be close to zero. Consistent with the function of insulators as genic barriers, one would not expect their deployment between non-transcribed loci—indeed what we observed (Supplementary Table S5). An important conclusion from all this is that if insulators separate genes then they could serve to define genes as well (see below).

When analyzing all vlincRNA–gene pairs across all distance bins, separation by insulators clearly reduced the positive correlation between vlincRNAs and adjacent genes on the same strand, consistent with the genomic barrier function of the former (Supplementary Table S4). Separation by insulators also reduced the positive correlation for the head-to-head vlincRNA–gene pairs on the opposite strand, while having little effect for the tail–tail pairs (Supplementary Table S4). However, even when separated by insulators, vlincRNA–gene pairs always had higher correlations than gene–gene pairs in the corresponding genomic configuration and distance range (Supplementary Table S4, Table 4). These differences in correlations between vlincRNA–gene and gene–gene pairs were highly statistically significant in all configurations, including vlincRNAs and genes located on opposite strands (Table 4).

Strikingly, however, insulators did not reduce the highest correlations between vlincRNA–gene pairs found at close distances (Supplementary Table S4). For example, while the presence of insulators almost nullified correlation between adjacent divergent head-to-head gene–gene pairs (0.315 versus 0.0415, Supplementary Table S4), the presence of insulators did not have the same effect on such vlincRNA–gene pairs. In fact, adjacent divergent head-to-head vlincRNA–gene pairs separated by insulators had higher correlation than pairs not separated by insulators (0.28875 versus 0.495, Supplementary Table S4). Assuming that insulators work in the same manner on all adjacent divergent transcripts (coding and non-coding), this observation implies that there are specific effects of vlincRNAs on adjacent genes that cannot be explained by shared chromatin environment. Moreover, the strong activating effect was also seen in the sense vlincRNA–gene pairs separated by relatively short distances and it was not reduced by insulators (0–5 kb, Supplementary Table S4). The effect was also observed in the tail-to-tail vlincRNA–gene pairs (correlation range of 0.11–0.10), but it never reached levels as high as in adjacent vlincRNA–gene pairs of the other three configurations (correlation range of 0.36–0.495) (Supplementary Table S4). In fact, the difference in vlincRNA–gene and gene–gene correlation in pairs separated by insulators decreased with increased distance but always stayed positive in ‘all’ configurations even at distance of >50 kb (Supplementary Table S3, Table 4). These results argue that the activating effect on nearby genes is a property of vlincRNAs in all configurations and even at longer genomic distances.

### vlincRNAs represent novel human genes

Using these characteristics of insulators, we proceeded to define standalone ncRNA genes from vlincRNAs. A vlincRNA achieved standalone gene status if it had no gene on the same strand within 50 kb (from either its 5′ or 3′ end) or if it was separated from any nearby gene by an insulator. In this scenario, a standalone vlincRNA has to be separated by an insulator from ‘all’ genes within 50 kb on the same strand. Finally, we collapsed the intervals to obtain 1542 unique novel ncRNA genes (Supplementary Table S6), representing ∼72% of 2147 original unique vlincRNAs described previously (3).

Moreover, 722 (46.8%) of the 1542 could be assigned to a promoter further supporting their status as standalone genes. However, the promoter dataset used in this work comes from a limited number of tissues and thus likely does not contain promoters for all vlincRNAs. Therefore, we supplemented this analysis by the FANTOM CAGE clusters representing enriched in sites of transcription initiation from the 833 tissues. As expected, a significant number of additional 256 out of 1542 (16.6%) vlincRNA genes had a CAGE cluster within 5 kb of annotated 5′ end. The remaining 564/1542 (36.6%) of vlincRNAs could still represent transcripts regulated by normal RNA Pol 2 promoters and the failure to annotate them as such could be due to technical issues for example their regulation by highly tissue-specific promoters or presence of repeated sequences within these promoters that would hamper their identification.

By definition, most of vlincRNA genes do not overlap with UCSC Genes on the same strand, however, genes shorter than 5 kb can be present inside boundaries vlincRNA (3). To estimate whether we are finding truly novel set of transcripts, we compared them with GENCODE v22 database. We searched for overlap of over 50% of vlincRNA gene length with an annotation to label a vlincRNA as present in those databases. The number of such overlapping vlincRNAs was 371/1542 (24.1%). These results show that most (75.9%) of the vlincRNA genes are novel standalone genes.

We compared these vlincRNA genes with previously identified mouse macroRNAs (24). Of the 66 mouse macroRNAs, 60 could be mapped to the HG19 version of the human genome. Of the 1542 vlincRNAs, 15 overlapped the 60 mouse macroRNAs. The significance (*P*-value 0.004) of the overlap supports our earlier observations that vlincRNAs tend to be syntenically conserved between human and mouse supporting their functionality (3). However, the fact that almost all vlincRNAs are novel underscores the fact that genes encoding very long non-coding transcripts represent a prominent, still unexplored and under-appreciated feature of mammalian genome.

### LTR vlincRNA genes are controlled by the pluripotency transcription factors OCT4, NANOG, SOX2

The association between vlincRNAs and pluripotency described above encouraged us to explore regulation of these RNAs by TFs associated with maintenance of the pluripotent state. OCT4, NANOG and SOX2 TFs are involved in the maintenance of the pluripotent state of human ES cells (37). To investigate this, we took advantage of the ChIPseq data for OCT4, NANOG and SOX2 binding sites from the H9 ES cell line (15). We found that flanking (+/−5 kb) DNA regions around annotated start sites of vlincRNAs were significantly enriched in binding sites of each of the three TFs relative to random genomic regions (*P*-value <0.001, Supplementary Figure S6). This suggested that these transcripts are indeed regulated by these TFs.

**Figure 4.**
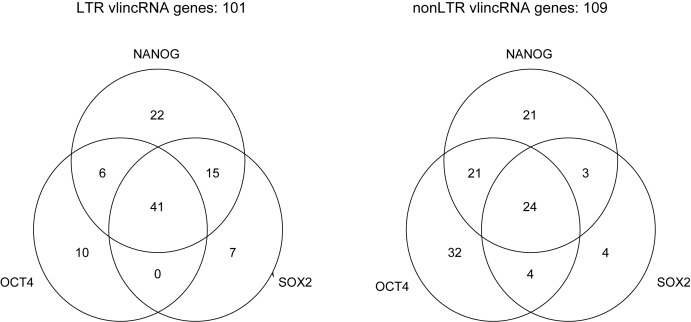
Fraction of each category of vlincRNA genes with binding sites for the pluripotency-associated TFs in the promoters. The number of LTR and nonLTR vlincRNA genes out of 1542 with the corresponding combination of TF binding sites at their promoters is shown.

**Figure 5.**
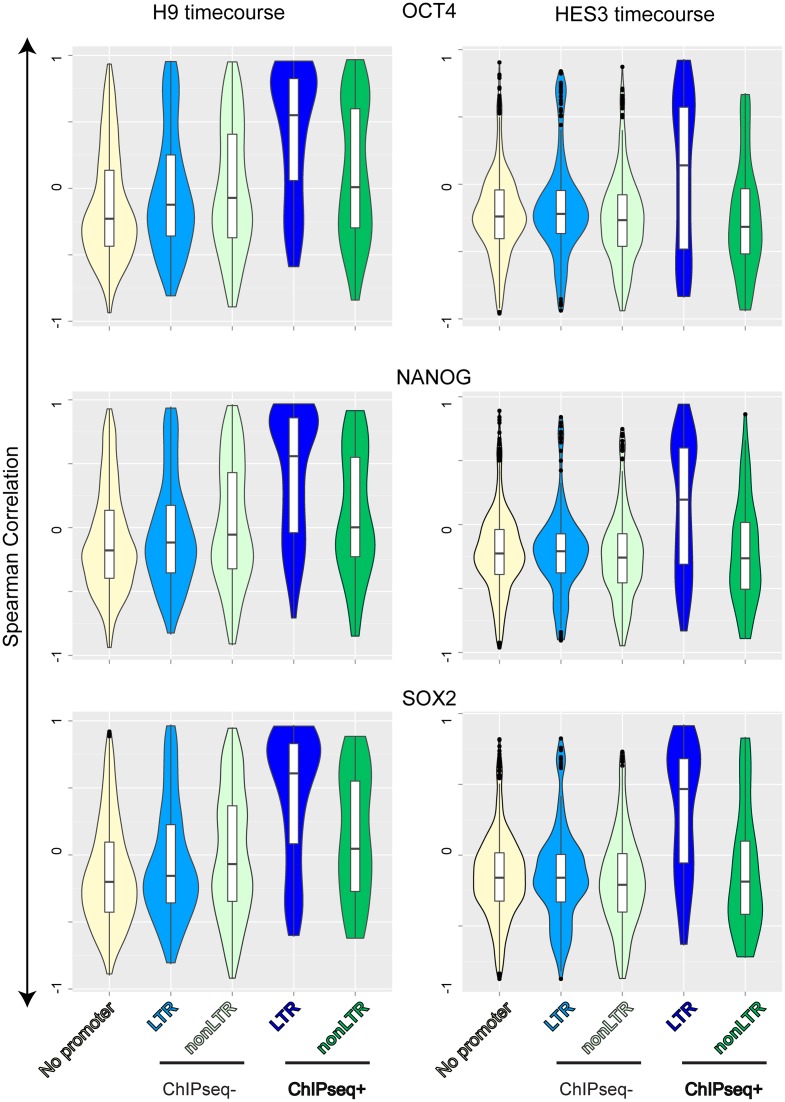
Distributions of Spearman correlations between levels of vlincRNAs and pluripotency-associated TFs. Spearman correlations were calculated between levels of each vlincRNA and each one of the three pluripotency TFs (SOX2, NANOG and OCT4) in the H1 (left) and HES-GFP (right) ESC differentiation timecoures. Violin plot distributions of these correlations are shown for different groups of vlincRNAs based on the presence of LTR (LTR and nonLTR) or ChIPseq signal (‘ChIPseq+’ or ‘ChIPseq-’) in their promoters. VlincRNAs without promoters are also included as controls.

To explore this in more details, we used the time course of H9 or HES3 ES differentiation CAGE FANTOM5 dataset (9). We first identified vlincRNAs associated with promoters which overlap binding sites for these TFs (15) as described in ‘Materials and Methods’ (Figure 4). If a TF regulates vlincRNA promoters to which it binds, then correlation of its expression level with the level of the corresponding vlincRNAs would be higher than with levels of vlincRNAs to promoters of which it does not bind. To test this, we calculated the Spearman correlation of expression of each vlincRNA with expression of each of the three TFs in the time course of H9 or HES3 ES differentiation. We then compared distribution of correlations between vlincRNAs whose promoters have and do not have evidence of TF binding. The 722 promoter-associated vlincRNA genes were also subdivided into ‘LTR’ and ‘nonLTR’ categories based on the presence or absence of LTR sequence in their promoters. Thus, for each TF we had five categories vlincRNA genes: (i) the ones that could not be assigned to a promoter; (ii) LTR and (iii) nonLTR vlincRNAs without the TF binding (the control group); (iv) LTR and (v) nonLTR vlincRNAs with the TF binding. The distribution of these correlations for each TF and different groups of vlincRNAs is shown in Figure 5 and the corresponding *P*-values are given in Supplementary Table S7.

We found a surprisingly high correlation between the expression levels of LTR vlincRNAs bound by each of the three TFs and the levels of these TFs in both time courses (Figure 5). The correlation for this group of vlincRNAs was substantially higher than all other groups— vlincRNAs without promoters, LTR or nonLTR vlincRNAs without the binding sites and the nonLTR vlincRNAs with the binding sites (Figure 5 and Supplementary Table S7). This was the case for each TF and in each of the two ES cell lines (Figure 5, Supplementary Table S7). The correlation for nonLTR vlincRNAs bound by TFs was also sometimes significantly higher than for the non-bound control (Supplementary Table S7, *P*-value < 0.05, one-sided KS), but the statistical significance was much lower than in the case of the LTR vlincRNAs (Supplementary Table S7). Because the vlincRNA and promoter datasets originated from different cell lines, conceivably some vlincRNAs without assigned promoters might also be regulated by the three TFs. However, this fraction has to be small because the correlation in this group of vlincRNAs was not significantly different from the control group (Figure 5, Supplementary Table S7).

These results indicate that levels of nonLTR vlincRNAs are primarily controlled by other means than the levels of the three TFs, yet the levels of LTR vlincRNAs in a significant measure depend on the levels of these TFs. Moreover, the correlation with the pluripotency-associated TFs cannot be explained by the general decrease of all LTR vlincRNAs in the process of differentiation of ES cells (Figure 2), because the control LTR vlincRNAs without the TF binding sites did not exhibit the high level of correlation (Figure 5, Supplementary Table S7). We therefore will refer to the 101 LTR vlincRNA genes bound by at least one of the three TFs as ‘pluripotency-associated vlincRNAs’.

**Figure 6.**
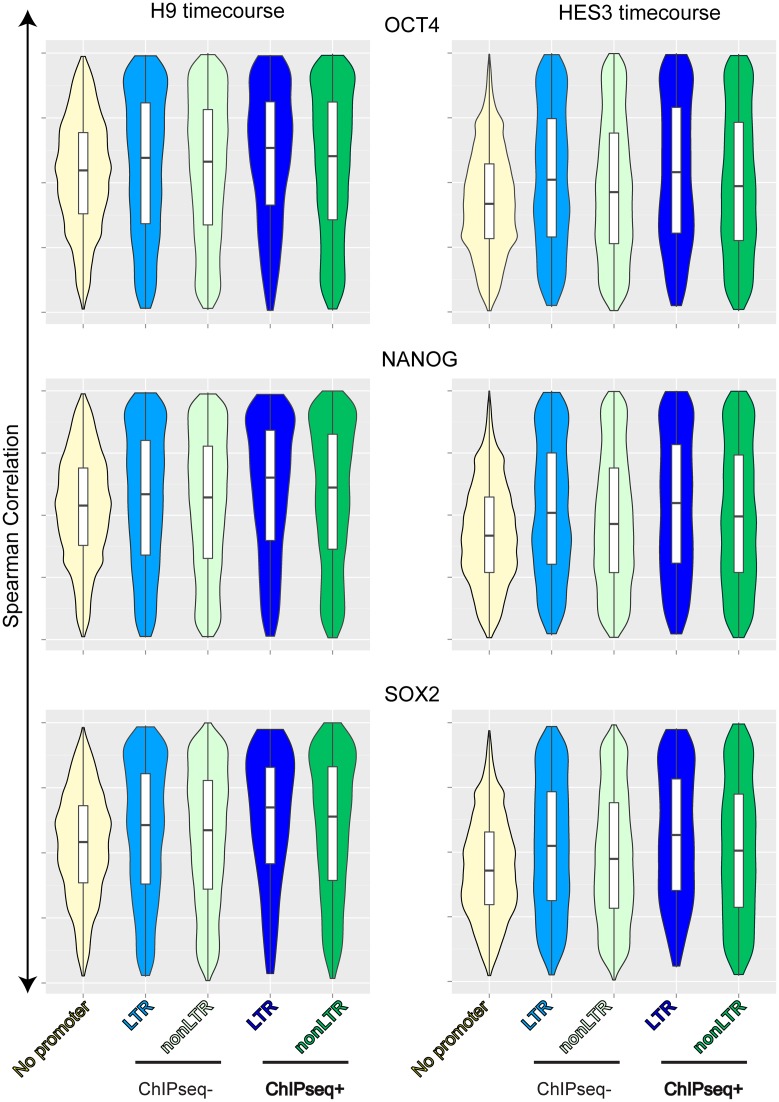
Distributions of Spearman correlations between levels of Known Genes and pluripotency-associated TFs. Spearman correlations were calculated between levels of each UCSC transcript and each one of the three pluripotency TFs (SOX2, NANOG and OCT4) in the H1 (left) and HES-GFP (right) ESC differentiation timecoures. Violin plot distributions of these correlations are shown for different groups of transcripts based on the presence of LTR (LTR and nonLTR) or ChIPseq signal (‘ChIPseq+’ or ‘ChIPseq-’) in their promoters. UCSC transcripts without promoters are also included as controls.

**Figure 7.**
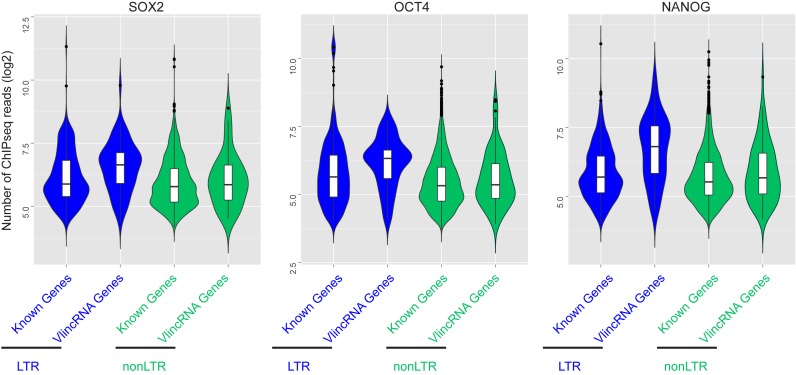
Distributions of ChIPseq signal in LTR and nonLTR promoters of vlincRNAs and Known Genes. Violin plots of the number of ChIPseq reads (log_2_ scale, Y-axes) for each of three pluripotency TFs in the different categories (LTR and nonLTR) of vlincRNA and UCSC Gene promoters are shown. For the latter, promoters with unique coordinates were used in cases when one promoter could be assigned to different transcripts. Only promoters that had at least one ChipSeq read were used for this analysis.

We then repeated the same analysis with promoters of known genes with and without LTR elements. Strikingly, the Spearman correlation distribution for the known genes was different than for vlincRNAs: the genes with the LTR and TF binding sites in the promoters did not exhibit a striking shift towards higher correlation observed in the corresponding vlincRNAs (Figures 5 and 6). Indeed, LTR ChIPseq+ vlincRNAs had a statistically significant shift towards higher correlations with the mRNA for the three TFs compared to nonLTR vlincRNAs and LTR or nonLTR genes with binding sites (Supplementary Figure S9). The reason for this became apparent however when we plotted the strength of ChIPseq signals at various promoters of vlincRNAs and genes in the H9 ES cell line (Figure 7). It revealed a much stronger signal at the promoters of LTR vlincRNAs for each of the three TFs compared to nonLTR vlincRNAs and LTR or nonLTR promoters of known genes (Figure 7). This apparent strong interaction argues for a stronger level of control of LTR vlincRNAs by the TFs and thus perfectly explains the much higher correlation between the expression levels of TFs and LTR vlincRNAs, which bind the TFs, compared to any other group of vlincRNAs or genes.

### Patterns of vlincRNA functionality based on co-expression with annotated genes

If a vlincRNA functions in a certain process, it is logical to suggest that it is also co-expressed with RNAs encoding other known functions involved in this process. Furthermore, some of these co-expressed RNAs might encode proteins (or other ncRNAs) that regulate vlincRNAs (like the pluripotency-associated TFs) or these RNAs represent targets of vlincRNAs themselves. Reciprocally, patterns of co-expressed genes can reveal what processes vlincRNAs could potentially be involved in. Therefore, we undertook the next logical step to functionally annotate vlincRNAs by examining whether vlincRNAs had a tendency to correlate with certain biological functions or certain biological processes (summarized in Supplementary Figure S7). As the first step, for each of the 1542 vlincRNA genes, we found annotated protein-coding transcripts positively or negatively correlating with it in the 833 tissues with Spearman correlation with >0.35 or <−0.35 respectively. We then looked for enrichment of certain biological functions or processes among these protein coding transcripts for each vlincRNA (Supplementary Figure S7). We recorded GO terms enriched with unadjusted *P*-value < 0.05, thereby establishing a list of GO terms for each individual vlincRNA. As a next step, we calculated enrichment of GO terms in particular subgroups of vlincRNAs such as the ‘pluripotency-associated vlincRNAs’ relative to the terms associated with all 1542 vlincRNA genes. Such GO terms would be expected to represent the molecular functions that a particular group of vlincRNAs correlates with, positively or negatively.

This analysis revealed some interesting findings as shown in detail in Supplementary Table S8 which lists the top 10 GO terms for each category of vlincRNA. Most importantly, the 101 pluripotency-associated vlincRNAs genes (LTR vlincRNAs with binding site for at least one of the pluripotency TFs) had a strong positive correlation with GO terms representing early embryonic processes. For example, ‘anterior/posterior axis specification’, ‘primitive streak formation’, ‘gastrulation with mouth forming second’, ‘midbrain development’, ‘axis specification’ and ‘gastrulation’ had adjusted *P*-values ranging from 10^−4^ to 4.4 × 10^−8^ (Supplementary Table S8). This is consistent with the involvement of this category of vlincRNAs in embryonic development as revealed above in the ESC differentiation time course and their regulation by the pluripotency TFs. It also validated this approach to the inference of vlincRNA functionality. This result can not be explained by regulation of vlincRNAs and genes in the above categories by the same three pluripotency factors. Indeed, while the RNA levels of ChIPSeq+ vlincRNAs and UCSC Genes do correlate with those of the three TFs in the stem cell differentiation time courses as shown above, this correlation disappears once all 833 samples are included (data not shown). On the other hand, the relationship with the early embryonic GO categories was calculated based on the entire set of 833 samples.

The nonLTR vlincRNA genes with the binding sites of pluripotency TFs also had an interesting pattern of positively correlating functions that could be classified as ‘cellular-level’ development functions that deal with cellular proliferation, migration and apoptosis rather than ‘embryonic-level’ functions found for LTR vlincRNAs. Cellular-level functions are represented by such terms as ‘positive regulation of cell development’, ‘stem cell proliferation’, ‘regulation of locomotion’, ‘apoptotic process’ and ‘cerebral cortex cell migration’ enriched with adjusted *P*-values ranging from 5.5 × 10^−6^ to 2.4 × 10^−6^ (Supplementary Table S8). In this respect, it is noteworthy that the nonLTR vlincRNAs also had strong negative correlation with cellular adhesion categories (Supplementary Table S8), consistent with the positive categories involved in cellular migration and locomotion. On the other hand, the general enrichment of GO terms in vlincRNAs (LTR or nonLTR) without the pluripotency TF binding sites was much less significant than in the ones with the sites (Supplementary Table S8). One potential reason for it is that the vlincRNAs without binding sites for a particular pluripotent TF with known function could likely represent mixtures of vlincRNAs with different functions. This underscores the enrichment of the specific functions of vlincRNAs with pluripotency TF sites. It is therefore likely that extending such analysis to other TFs would likely reveal additional functional patterns of remaining vlincRNAs.

To exclude a possibility that the above mentioned results could be explained by expression of other genes in the vicinity of vlincRNAs rather than vlincRNAs themselves, we repeated the GO analysis with vlincRNA genes that do not have a UCSC gene within 50 kb of either strand. The results obtained with 39 (out of 101) LTR ChIPSeq+ vlincRNAs confirmed previous results. Of the top 10 GO terms, 6 were associated with early embryonic development: ‘anterior/posterior axis specification’, ‘primitive streak formation’, ‘gastrulation with mouth forming second’, ‘gastrulation’, ‘axis specification’ and ‘midbrain development’ with the corresponding adjusted *P*-values 1.89 × 10^−6^, 6.69 × 10^−6^, 1.54 × 10^−5^, 2.13 × 10^−5^, 6.97 × 10^−5^ and 1.13 × 10^−4^. The corresponding number of nonLTR ChIPSeq+ vlincRNAs was too small for a meaningful *P*-value analysis. Nonetheless, these results argue that the observed associations are due to expression of vlincRNAs per se rather than other genes in their genomic surroundings.

### Majority of ‘pluripotency-associated vlincRNAs’ has not been previously associated with this function

Activation of ncRNA derived from retrotransposon promoters and enhancers has been previously reported by us (3) and other groups (23,27). Specifically, aided by focusing CAGE analysis of nuclear fraction rich in non-coding transcripts (38), Fort *et al*. have identified a class of ‘NASTs’ involved in maintenance of pluripotency in human and mouse ESC (23). We have therefore investigated how many of our vlincRNA genes overlapped with the NAST CAGE clusters. As shown in Table 5 and Supplementary Figure S8, 7.3% (53 out of 722) vlincRNA genes had a promoter with a NAST CAGE cluster. However, as expected considering the ESC-specific nature of NASTs, this fraction increased to 22.8% (23 out of 101) for the 101 ‘pluripotency-associated vlincRNAs’ (LTR ChIPseq+). In addition, 10.1% of nonLTR ChIPseq+ vlincRNAs had a promoter with NAST. Also, as expected, the fraction of ChIPseq- vlincRNAs that had NAST clusters in their promoters was significantly lower (Table 5, Supplementary Figure S8).

**Table 5.** Overlap with NAST CAGE clusters

		Overlap in promoters			Overlap in promoters and gene bodies		
	VlincRNA genes in each category	Overlapping with NASTs	Overlapping with NASTs, %	*P*-value^a^	Overlapping with NASTs	Overlapping with NASTs, %	*P*-value^a^
LTR Chipseq+	101	23	22.77%	1.18E-145	35	34.65%	2.88E-199
LTR Chipseq-	185	9	4.86%	5.16E-10	19	10.27%	1.66E-01
NonLTR Chipseq+	109	11	10.09%	3.98E-25	17	15.60%	9.54E-23
NonLTR Chipseq-	327	10	3.06%	4.23E-13	25	7.65%	1.81E-01
With promoters	722	53	7.34%	1.91E-159	96	13.30%	3.15E-98
Total	1542				130	8.43%	1.29E-57

^a^*P*-values based on Fisher's exact test from Bedtools suite.

Since CAGE clusters could also occur within bodies of transcripts, we then extended the analysis to include promoters and internal regions of vlincRNAs (Table 5). Predictably, the overlap increased and the ‘pluripotency-associated vlincRNAs’ still had the highest fraction of association with NASTs: 34.7% (35/101). Overall, 13.3% (96/722) of vlincRNA genes with assigned promoters overlapped a NAST cluster in a promoter or a body of the transcript (Table 5, Supplementary Figure S8). Finally, 8.4% (130/1542) of all vlincRNAs genes had a NAST cluster either in promoter or gene body. While association of every group of vlincRNA gene with NAST cluster was significant either in promoters or in promoters + gene bodies (Table 5, Supplementary Figure S8) as would be expected for bona fide transcripts detected by multiple methods, the most significant association was seen with the ChIPseq+ vlincRNAs, especially the LTR-driven ones. Still, this work revealed the association of ∼2/3 of the 101 ‘pluripotency-associated vlincRNAs’ with this biological function.

### vlincRNAs localize in the nucleus in a punctate pattern

**Figure 8.**
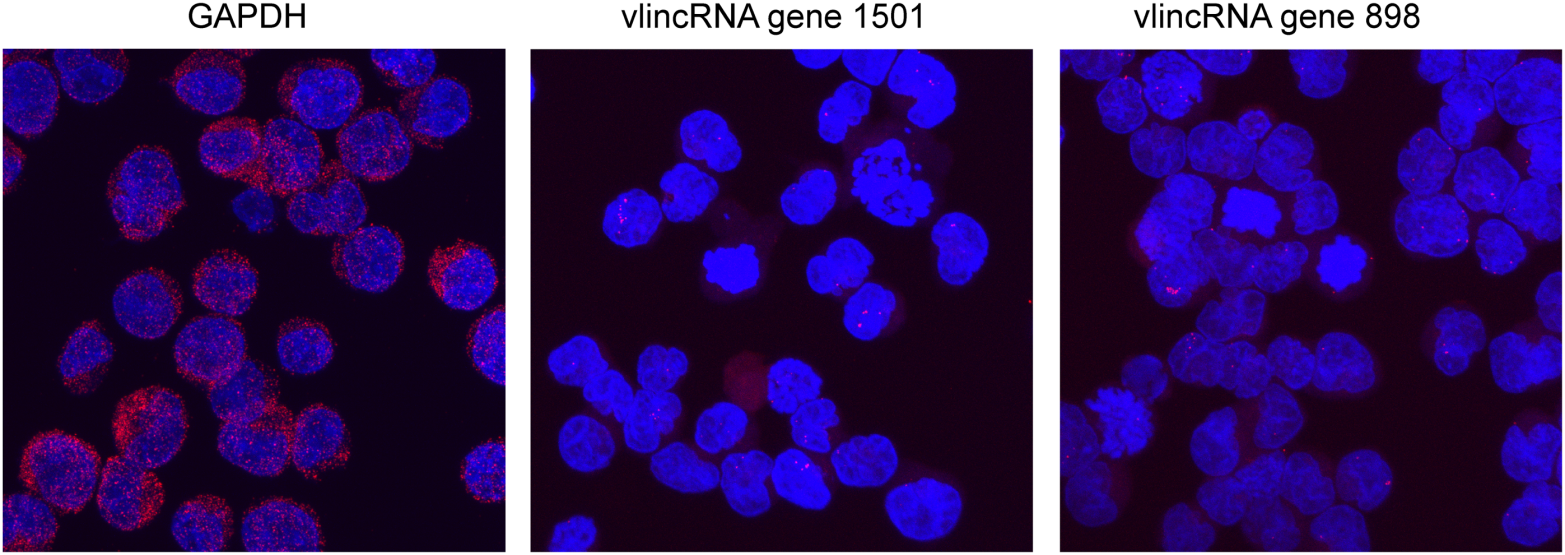
*In-situ* localization of vlincRNAs using RNA-FISH. K562 human erythroleukemia cells were fixed and analysed by RNA-FISH for with probes against GAPDH and two LTR vlincRNAs vlinc_377 and vlinc_500 originally identified in (4) corresponding to vlincRNA genes with IDs 1501 and 898 correspondingly in Supplementary Table S6. RNA probes were labelled with ATTO red (red), hybridized and washed extensively prior to counterstaining of DNA with DAPI (blue). Confocal microscopy was used to obtain z-stacks of images that were then digitally flattened, and then the red and blue channels were merged to produce the composite image.

Not much is known about the mechanisms of vlincRNA function. The only well-characterized member of this class is VAD vlincRNA involved in maintenance of cellular senescence (6). VAD modulates chromatin structure in *cis* and *trans* and also affects expression of specific genes in *trans* (6). Results presented here also suggest that vlincRNAs could function in *cis* and potentially in *trans* based on their correlation with nearby and distal genes. We wondered whether vlincRNAs reported here and VAD might share additional properties that might indicate common functional mechanisms and provide additional evidence for grouping these transcripts into one class. One such property critical for RNA function could be subcellular localization. VAD, the only vlincRNA for which subcellular localization data is available, has a fairly distinct punctate nuclear localization pattern (6). Therefore, we chose two LTR vlincRNA genes (#898 and #1501, Supplementary Table S6) for localization in K562 cells using RNA fluorescent *in situ* hybridization (FISH) using RNAscope technology (25) (Figure 8). Both vlincRNAs exhibit a highly localized punctate pattern in the nucleus, similar to that of VAD (Figure 8). Overall, ∼95% of the puncta for the vlincRNAs were nuclear, supporting nuclear localization of these RNAs, unlike the signal from GAPDH probe where the majority of the dots were outside of the nucleus (Figure 8). The two vlincRNAs were expressed in ∼60% cells (63% #1501 and 58% #898), similar to senescence-associated VAD expressed in 73% of senescent cells (6). Among the expressing cells, the number of dots per cell ranged from 1 to >10, with the largest number of expressing cells (∼40%) having just one dot. However, 44.3% of cells had three dots or more similarly to the VAD vlincRNA which could also be localized to multiple (three or more) sub-nuclear locations in 16% of cells (6). Using the DAPI staining pattern of the nuclear DNA (blue), overlaid with the vlincRNA FISH (red), it is obvious that the punctate nuclear pattern of vlincRNA staining is lost in cells that are in the DAPI-bright metaphase to telophase stages. Further, in non-dividing cells, the vlincRNA punctate staining was almost invariably located to DAPI-dark euchromatic regions, suggesting that it emanates from or rapidly localizes to transcriptionally active regions. In summary, at least three vlincRNAs—two reported here and VAD (6)—appear to localize in the nucleus with a similar distinct punctate pattern suggesting potential general similarities in the mechanisms of action of at least some transcripts of this class. However, additional vlincRNAs would need to be tested to determine whether this is a common property of these transcripts.

## DISCUSSION

The place occupied by the vlincRNAs within the genome's architecture and their functional parameters constitute perhaps the most important questions one could ask about this class of ncRNAs. We show that the majority (1542 out of 2147) of the vlincRNAs, even those adjacent to known genes, represent standalone ncRNA genes. This conclusion is based on several observations, but most notably by the analysis of insulators—genetic elements initially discovered as barriers separating genomic elements. While more recent work questioned the function of these elements (13), here we show that indeed insulators do act globally as genomic barriers and separate standalone genic units. Using separation by insulators from existing annotations as one of the main criteria, we found that vlincRNAs represent at least 1542 new genes. This includes the identification of hundreds of vlincRNAs (452 out of 1542) adjacent and on the same strand as known genes, yet representing separate genes.

Even when separated by insulators, vlincRNAs are positively correlated with the expression of adjacent or nearby genes on the same or opposite strands. As described above, this effect was most striking on the adjacent head-to-head pairs. Assuming that the basic transcriptional and chromatin machinery is the same for known genes and vlincRNAs, the effect of insulators would be the same on any pair of transcriptional units. Thus, the positive correlation could not be explained by the shared chromatin environment because vlincRNA–gene pairs separated by insulators always had higher correlation than gene–gene pairs. The local regulatory effect appears to peak at closer distances and manifest itself the most in sense and head-to-head configurations of vlincRNA–gene pairs. Overall, the data presented here appears to suggest a paradigm of regulation similar to the recently reported enhancer-like RNAs or activating ncRNAs (ncRNA-a's) (11,12). At present, we cannot totally exclude the possibility of the reciprocal effect whereby adjacent genes positively regulate vlincRNAs. However, by the analogy with the activating RNAs it is logical to suggest that vlincRNAs fulfil similar functions and activate nearby genes in *cis*.

The positive regulatory effect of vlincRNAs may not be limited to nearby genes. For examples, VAD vlincRNA participates in the activation in *trans* of the INK4 locus, which contains anti-proliferative genes required for the senescence-associated proliferation arrest (6). Inhibition of HELLP vlincRNA with siRNAs causes mostly downregulation of expression of target genes (39) suggesting positive regulation of its targets. This would be consistent with an idea of rather diverse modes of ncRNA function (40,41), including as intelligent scaffolds guiding various processes associated with regulation of gene expression in nucleus (42). In this case, the punctate pattern of nuclear localization could be revealing subnuclear domains where these vlincRNAs control expression of specific subset of genes.

Perhaps the most notable result revealed here is that a System Biology-based approach integrating different datasets could distinguish subsets of a novel class of ncRNAs—vlincRNAs—based on predicted biological function. One such prominent subset likely functions in early embryonic development and includes vlincRNAs with LTR promoters and binding sites of any one of the three pluripotency-associated TFs. While we do not yet have direct genetic evidence, the conclusion relies on four independent lines of investigation. First, expression of this group of vlincRNAs is highest in pluripotent ES cells and downregulated during their differentiation. Pluripotent cells are known to have more active genomes (43), however, if downregulation of the LTR vlincRNAs during ESC differentiation simply reflected general chromatin silencing, it is not clear why nonLTR vlincRNAs would not follow the same trend. Second, the vlincRNAs have upstream promoters with consensus binding sites for well-established pluripotency TFs. Likewise, there is very strong evidence of regulation of vlincRNAs by these TFs at least during the time course of ESC differentiation. While LTR vlincRNAs in general have a tendency to decrease during the time course of ESC differentiation, only those with the binding sites correlate strongly with the levels of these TFs. Furthermore, binding by the TFs per se does not necessarily equate with regulation, as shown by the nonLTR vlincRNAs. Thus, the presence of multiple features in their promoters—LTR and binding sites—defines the functioning and regulation of these ncRNAs. Third, co-regulation of these 101 novel vlincRNA genes in all 833 tissues with genes enriched in early embryonic functions provides additional strong support of their function. Finally, the promoters of the 101 LTR vlincRNA genes exhibit the strongest binding of each of the three TFs relative to other vlincRNAs and even more importantly, the known genes. Combined, these four lines of evidence argue against spurious, leaky association of vlincRNA with known transcripts, and are more consistent with highly regulated transcription of the vlincRNAs. However, additional experiments are required to completely rule out this possibility for all vlincRNAs.

These results add to the growing evidence that remnants of endogenous retroviruses have had important and far reaching impact on shaping our development as a species. The categories of genes correlating with pluripotency-associated vlincRNAs included early brain development with intriguing possibilities of involvement of these RNAs in defining species-specific brain functions as has been previously proposed (40,44).

The strength of associations with various functional GO terms for the 210 vlincRNA genes bound by the pluripotency TFs contrasts markedly with relatively low significance of associations for the 512 vlincRNA genes not bound by these factors. The likely scenario is that the latter category contains a mixture of various functionalities yet to be revealed by a comprehensive approach similar to this one. However, this provides a good control for the functional enrichment observed for the vlincRNAs with the binding sites. Interestingly, the 109 nonLTR vlincRNA genes bound by the pluripotency TFs had different patterns of functions. While also related to development, they positively correlated with cellular (including stem cell) proliferation, migration and apoptosis and negatively correlated with cell adhesion. This evidence supports our previous experiments that revealed a much higher fraction of apoptosis after inhibition of nonLTR vlincRNAs compared to LTR vlincRNAs (3).

Functionality of ncRNAs is the coveted goal of modern genetics research. However, as discussed by Mudge *et al*. (7), efforts to reach this lofty objective require systems biology approaches rather than traditional forward genetics methods from the protein-coding world. These approaches must also aim to achieve integration between distinct large genome-scale datasets, even with the inherent issues with noise (5). Here we show the feasibility of this approach by identifying potential functional properties for 210 new vlincRNA genes. This includes the 101 novel genes encoding non-coding RNAs potentially involved in early development representing a significant addition to our knowledge of molecular actors involved in this process. Additional experiments would be required to unequivocally prove their function and characterize mechanisms of action.

Overall, our results suggest that vlincRNAs represent a hitherto hidden layer of regulation involved in critical biological processes and disease, participating as yet un-appreciated molecular components of these processes. As we show here, well-characterized TFs could have additional facets of their function mediated by vlincRNAs. In addition, these ncRNAs could represent functional missing links that connect hits from genome-wide association studies to phenotypes (45).

## Supplementary Material

SUPPLEMENTARY DATA
