# Supplementary material for: Functional annotation of the vlinc class of non-coding RNAs using systems biology approach
Source: Nucleic Acids Res. 2016 Mar 21;44(7):3233–52. doi: 10.1093/nar/gkw162 (PMC4838384; doi:10.1093/nar/gkw162)
Supplement: SUPPLEMENTARY DATA [file supp_44_7_3233__index.html]

Functional annotation of the vlinc class of non-coding RNAs using systems biology approach — SUPPLEMENTARY DATA 

# Functional annotation of the vlinc class of non-coding RNAs using systems biology approach

## SUPPLEMENTARY DATA

- SUPPLEMENTARY DATA
- SUPPLEMENTARY DATA
- SUPPLEMENTARY DATA
- SUPPLEMENTARY DATA
- SUPPLEMENTARY DATA
- SUPPLEMENTARY DATA
- SUPPLEMENTARY DATA
- SUPPLEMENTARY DATA
- SUPPLEMENTARY DATA
