# Supplementary material for: Functional annotation of the vlinc class of non-coding RNAs using systems biology approach
Source: Nucleic Acids Res. 2016 Mar 21;44(7):3233–52. doi: 10.1093/nar/gkw162 (PMC4838384; doi:10.1093/nar/gkw162)
Supplement: SUPPLEMENTARY DATA [file supp_gkw162_nar-00129-z-2016-File010.pdf]

Supplementary Figure 1. Aggregate plots of CAGE or RNAseq signal in K562 vlincRNA gene bodies and in flanking regions. genomic interval corresponding to each of the 404 vlincRNAs originally found in the K562 cell line was divided in 100 bins (UCSC exons and rRNA repeats on either strand were excluded) (bins 101-200 on the X-axes). In addition, the 5' upstream 5kb and 3' downstream 5kb flanking regions of each vlincRNA were also subdivided in 100 bins (bins 1-100 and 201-300 correspondingly on the X-axes). Cumulative signal for CAGE (FANTOM5 sample "chronic myelogenous leukemia cell line:K562 ENCODE") (top) and SMS RNAseq (K562 cell line) (bottom) represents sums of all tags in each bin for each vlincRNA.

Supplementary Figure 2. Scatter plots of CAGE vs RNAseq signal for UCSC Known genes. For each of UCSC transcripts with exonic length over 1000 nt, both the CAGE and RNAseq RPKM expression values were obtained based on the "internal" counting within the exons. Both CAGE (Y-axes) and RNAseq (X-axes) expression values were converted to ranks and plotted for K562 (A) and whole blood (B).

Supplementary Figure 3. Sensitivity of detection of vlincRNAs by CAGE using internal or 5' flanking counting methods. For each vlincRNA from the indicated cell lines or tissues, CAGE counts were obtained either by counting within gene bodies ("internal") or within +/-5kb of the 5' ends ("5' flanking"). The histograms show numbers of vlincRNA (Y-axes) detected with the indicated number of tags (X-axes) for each method in each sample. For each sample, p-values of observing the higher sensitivity by chance (binomial test) are shown.

Supplementary Figure 4. Distribution of maximum, average and median values for cancer and normal tissues for LTR and nonLTR vllincRNAs. The maximum, average or median RPK100M values were calculated for each LTR or nonLTR vlincRNA in 399 normal (red) and 332 cancer (blue) tissues. The corresponding values were ranked and plotted on each graph.

Supplementary Figure 5. Box plots of the relative masses of LTR and nonLTR vlincRNAs in different tissue types. Relative masses of distal (>50kb from annotations on both strands) LTR (left) and nonLTR (right) vlincRNAs were calculated relative to total informative CAGE reads (top) or total vlincRNA reads (bottom) in cancer, immortalized, normal or stem tissues or cell lines. The plots were generated using R package and Supplementary Table 3 data.

Supplementary Figure 6. ChIPSeq sites for the 3 pluripotency TFs are enriched in vlincRNA genes promoter regions. Empirical cumulative distribution functions (ECDF's) represent cumulative distributions of the numbers of transcription factor binding sites (ChIP-Seq peaks) (X-axes) in 1542 randomly chosen non-overlapping 10 kb genomic sites. The random genomic regions selection was repeated 1000 times. ECDF of the resulting 1000 numbers of overlapping ChIP-Seq peaks for Oct4 (red), Nanog (yellow) and Sox2 (blue) are shown. For comparison, the observed counts of ChIP-Seq peaks found in the actual vlincRNA promoter regions (+/- 5kb around 5' ends) are plotted with dashed lines. The overlap with the random regions is always much smaller than with the actual vlincRNA promoters.

Supplementary Figure 7. Diagram of pipeline of the GO analysis of genes co-expressed with vlincRNAs.

Supplementary Figure 8. Summary of the overlap with NAST regions. The counts of overlapping NAST CAGE clusters with different categories of vlincRNAs are shown. Overlapping was done with bedtools suite based on hg19 genome coordinates.

Supplementary Figure 9. LTR vlincRNAs have higher correlation with the 3 pluripotency TF than nonLTR vlincRNAs and UCSC Genes. Each plot shows empirical cumulative distribution functions (ECDF's) of Spearman's correlation coefficients (X-axes) between expression values of mRNA for the indicated TF (NANOG, OCT4 or SOX2) and either LTR or NonLTR vlincRNAs or LTR or NonLTR UCSC Genes that have binding site for this TF in their promoters. The correlations were calculated for H9 (left) or HES3 (right) ESC differentiation time course based on the CAGE data. The p-values shown on each plot represent significance of ECDF of LTR vlincRNAs being lower than ECDF of NonLTR vlincRNAs, LTR and NonLTR UCSC Genes based on one-sided Kolmogorov-Smirnov test.

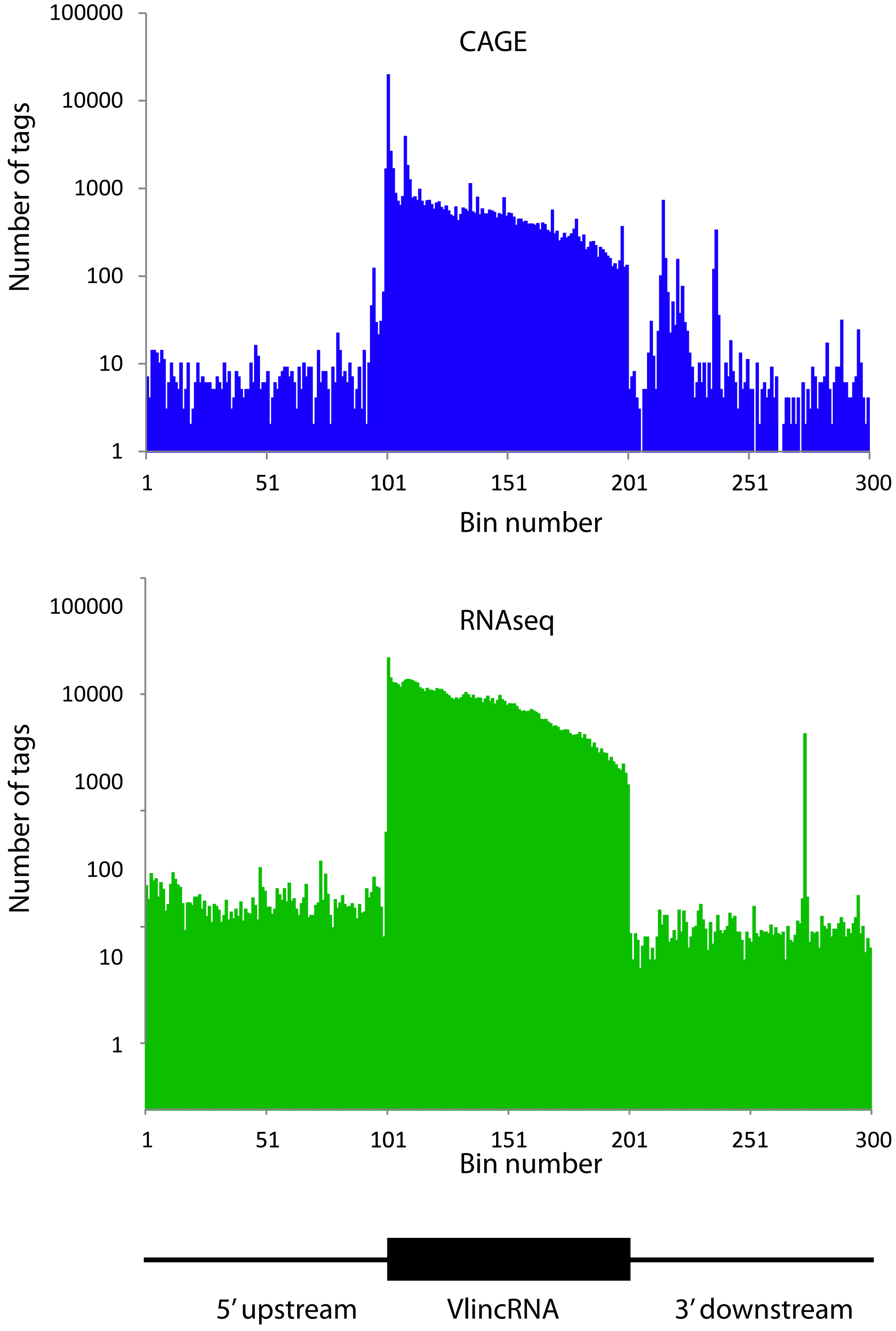

Supplementary Figure 1

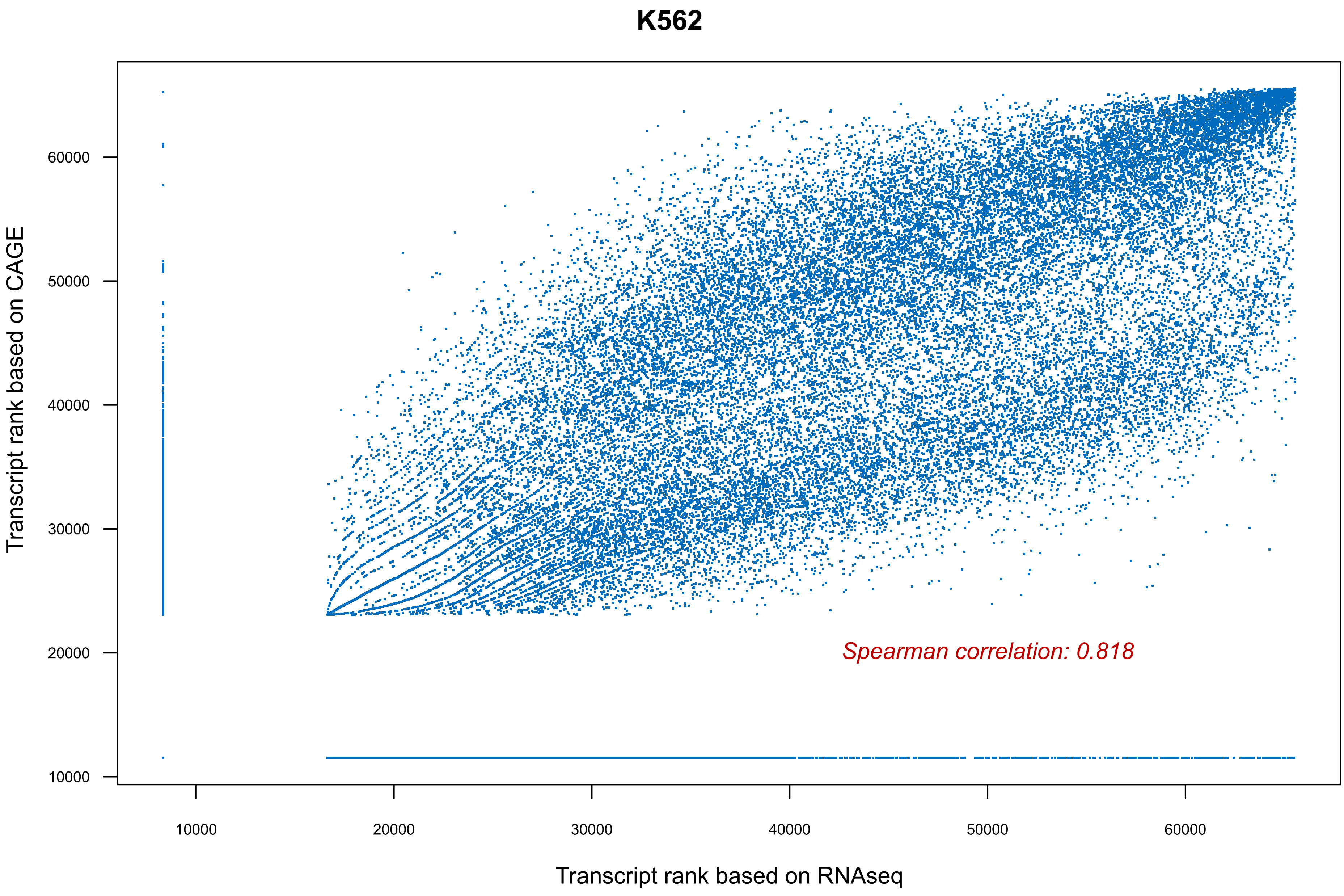

Supplementary Figure 2A

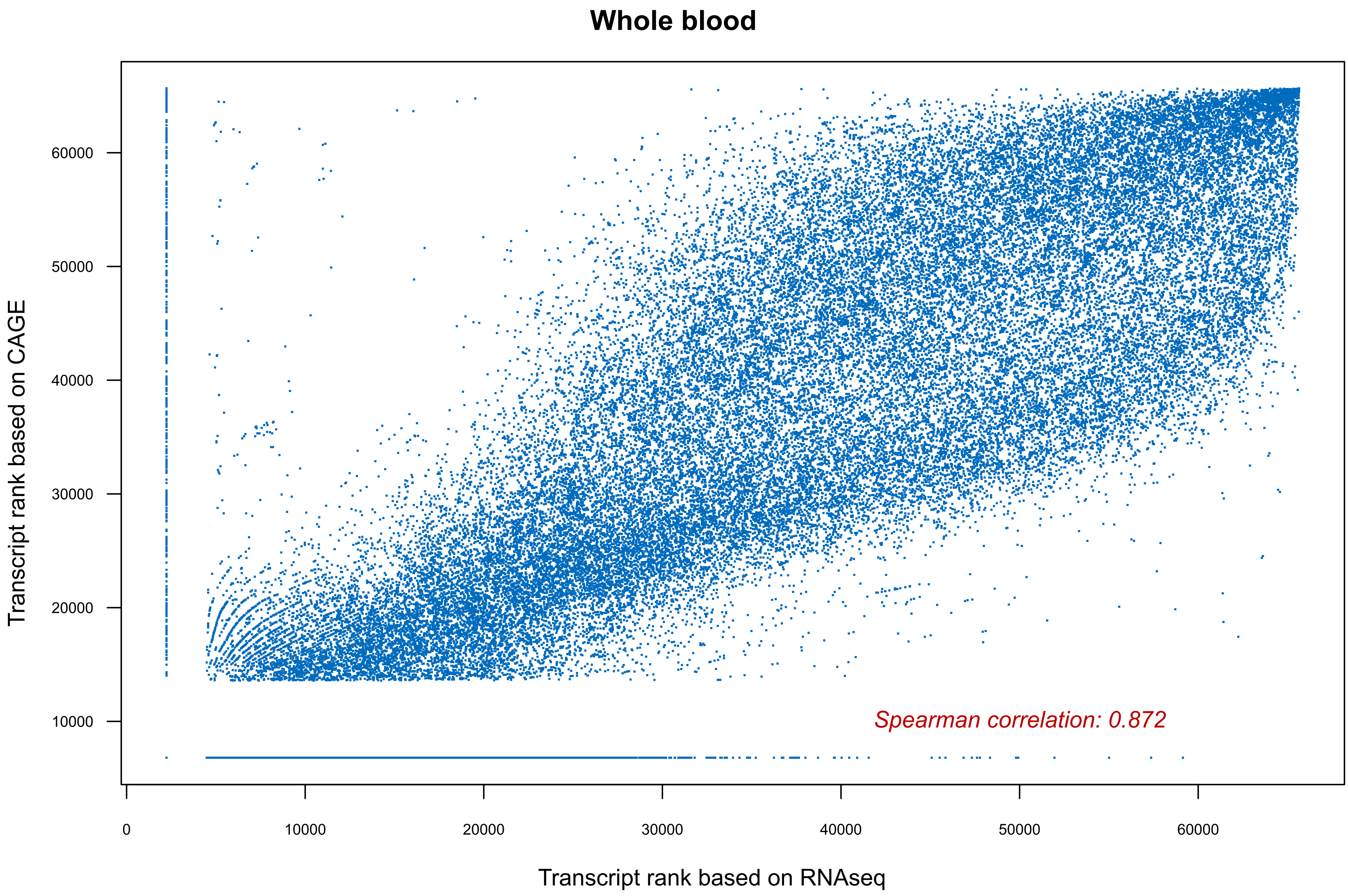

Supplementary Figure 2B

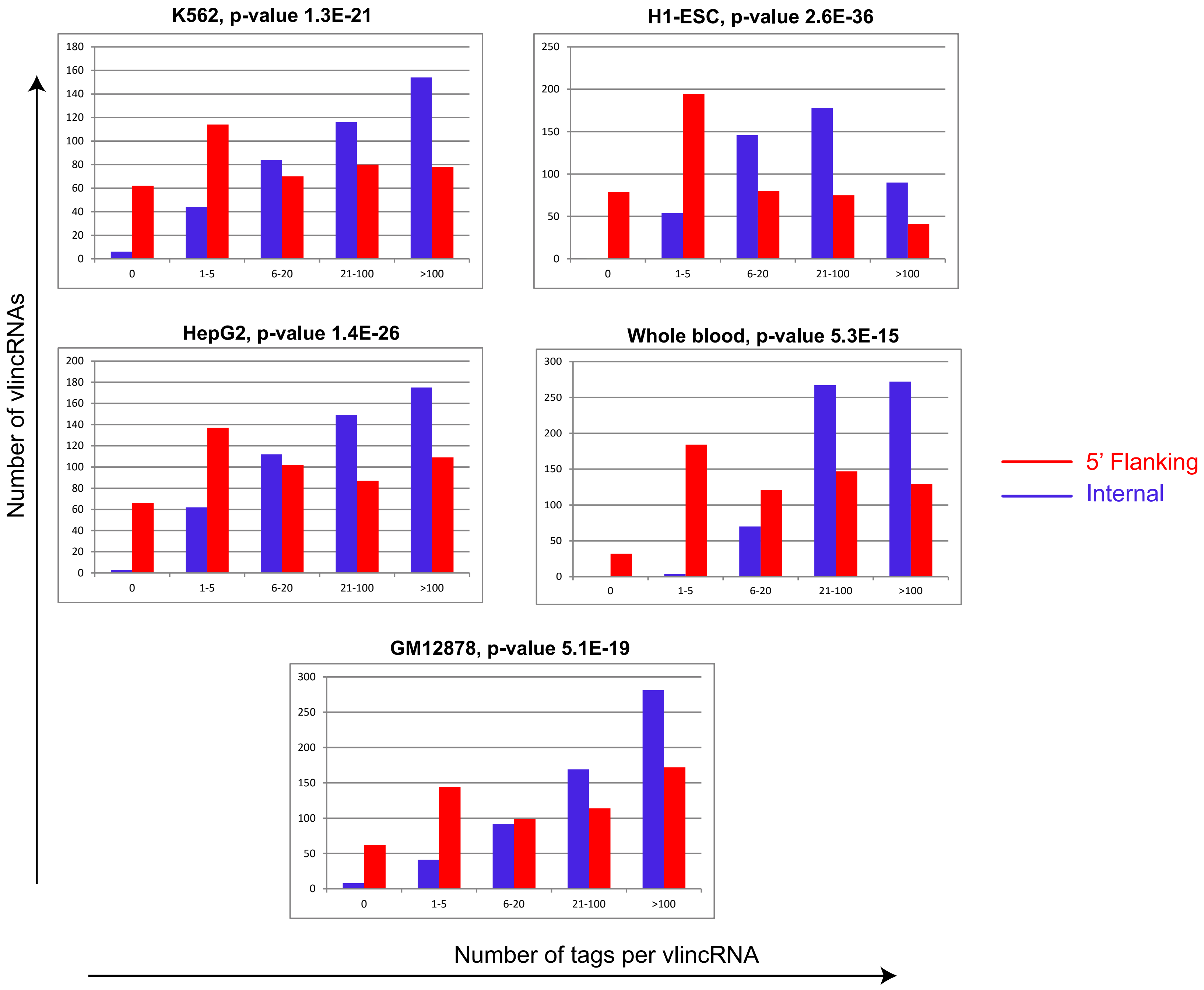

Supplementary Figure 3

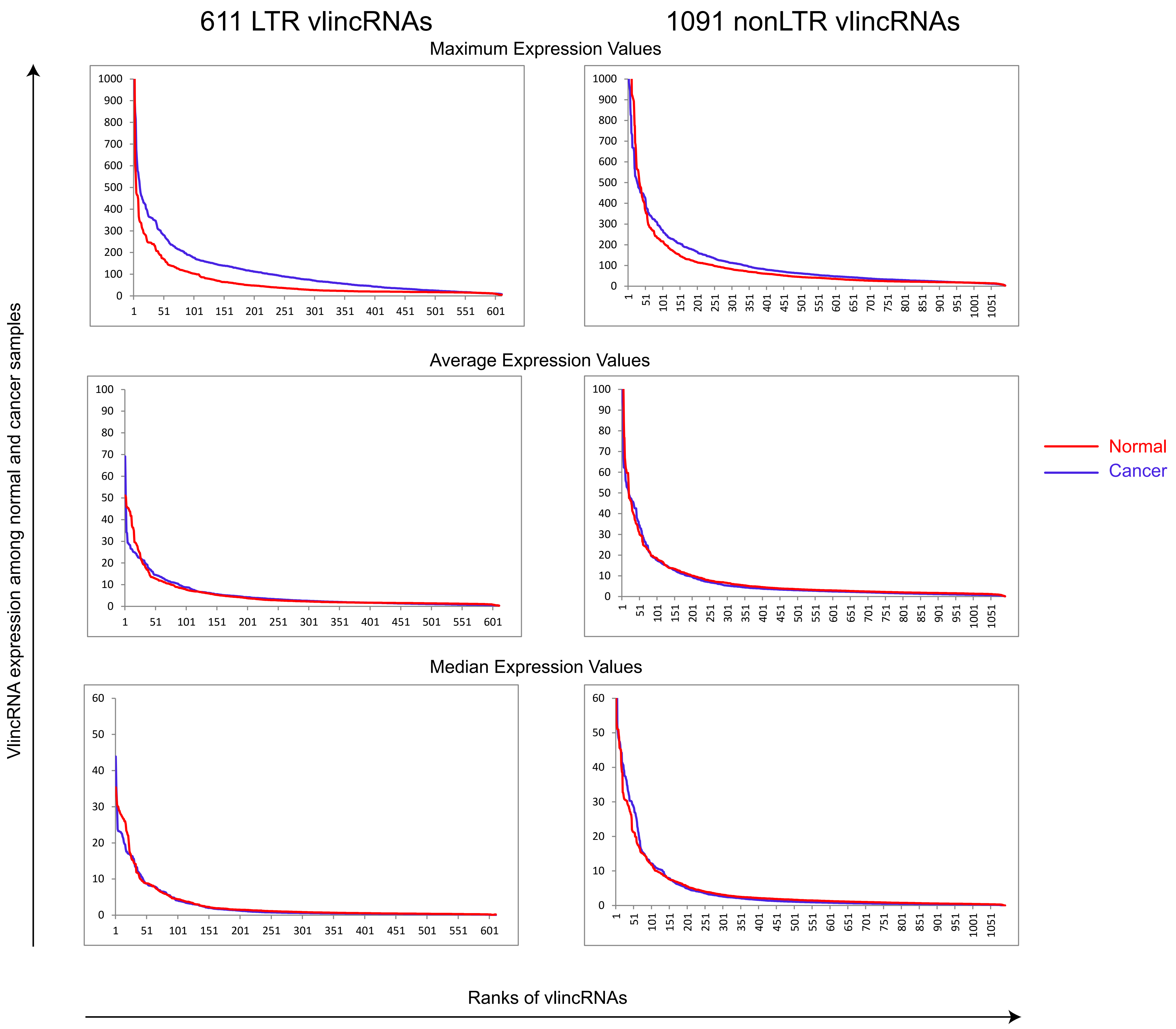

Supplementary Figure 4

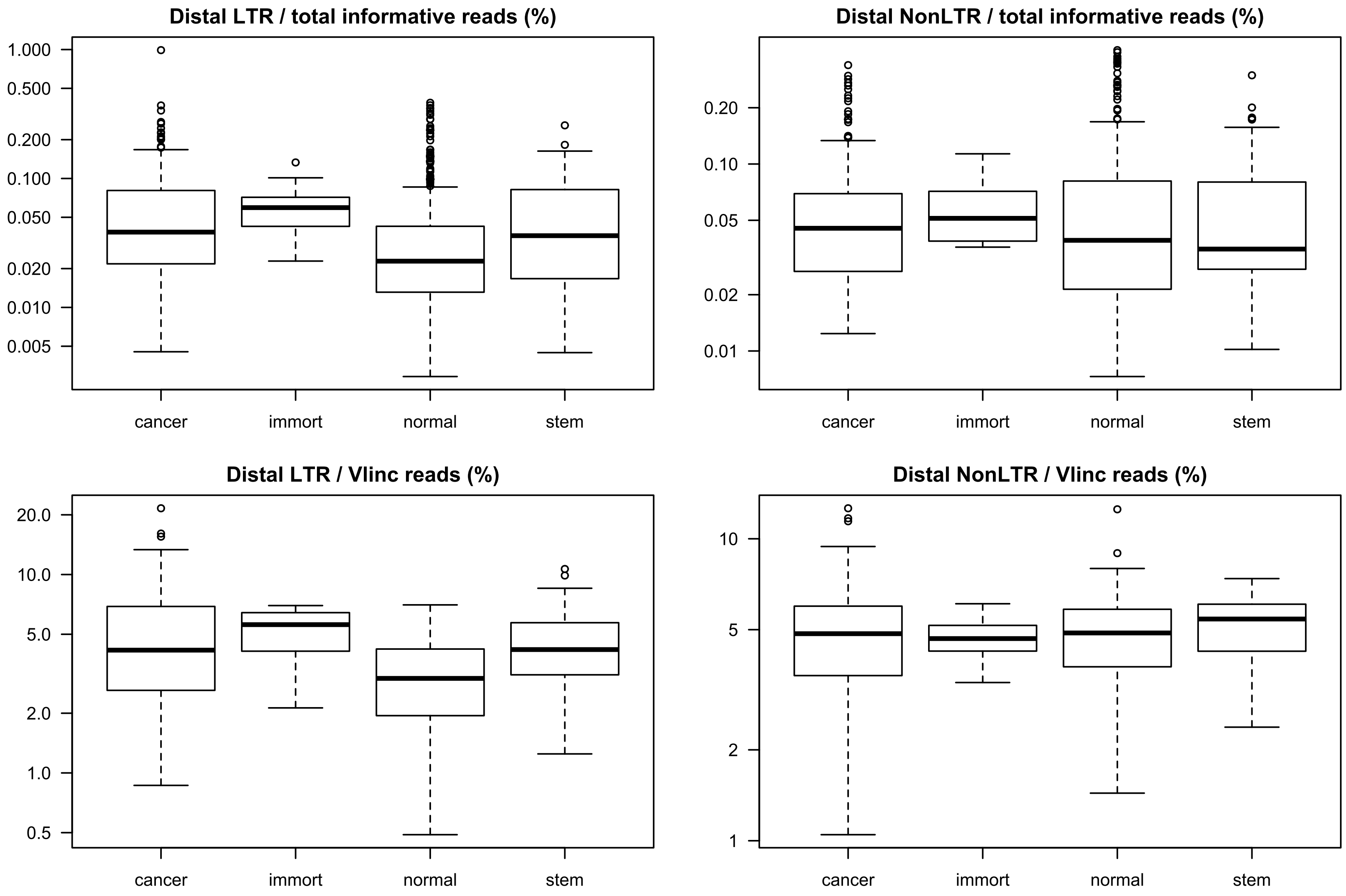

Supplementary Figure 5

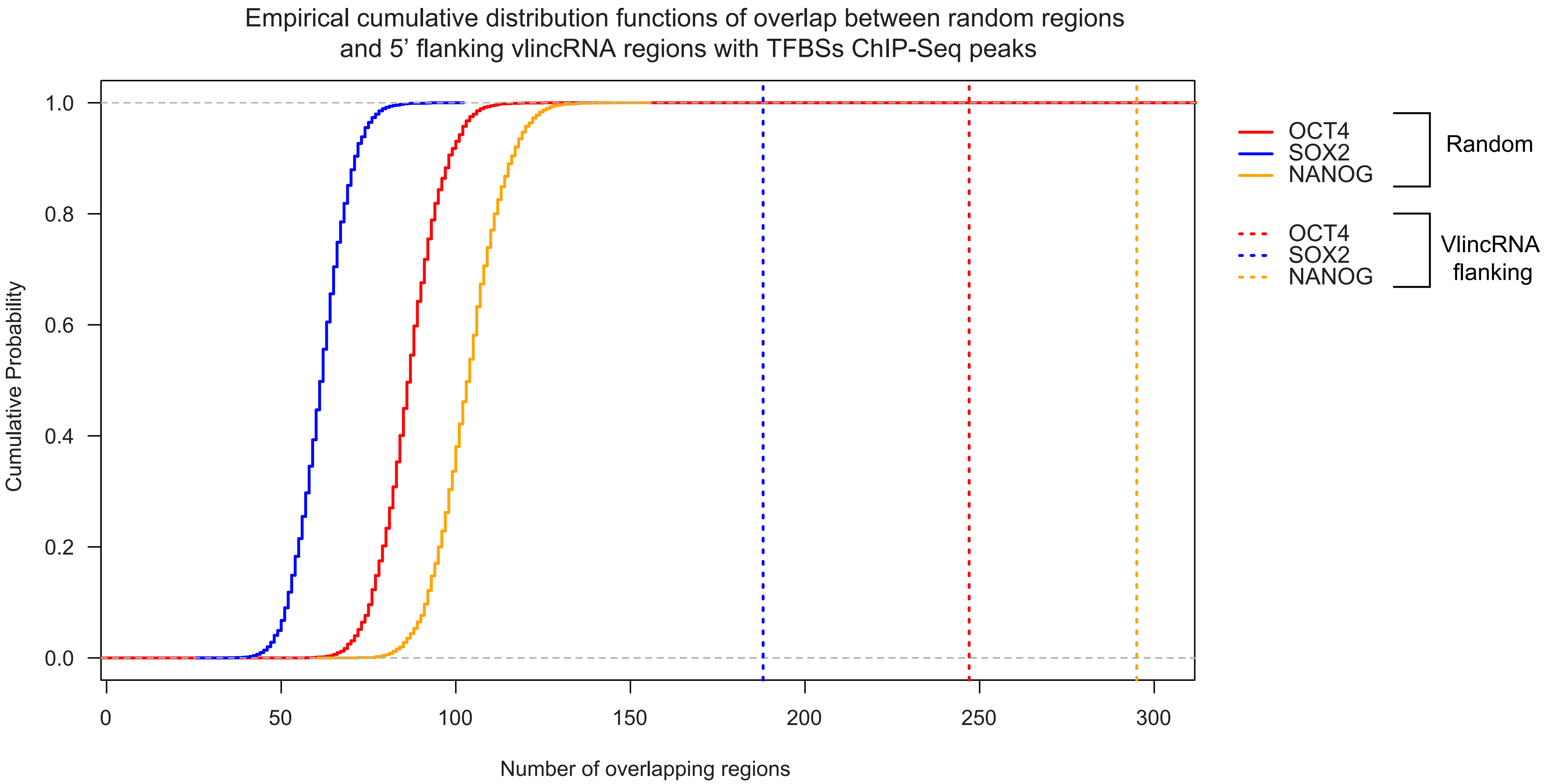

Supplementary Figure 6

Find annotated mRNAs that correlate with each standalone vlincRNA gene with Spearman correlation across all 833 tissues ≥0.35 or ≤-0.35

↓

Each vlincRNA gene out of 1542 is assigned to a list of genes positively or negatively correlating with it

↓

Enrichment of GO terms is calculated for each vlincRNA based on the total number of correlated (positively and negatively separately) genes with this GO term expected by chance

↓

Each vlincRNA is assigned to a list of GO terms positively or negatively correlating with it

↓

Select a subset of vlincRNAs, for example ChIPSeq+ LTR, and test whether certain GO terms are enriched in this subset relative to all 1542 vlincRNAs

↓

A list of GO terms enriched among genes positively or negatively correlating with a subset of vlincRNAs

Supplementary Figure 7

	Promoter	vlincRNA	Total	NAST cluster in promoter	NAST cluster in promoter or in a body of transcript
All			1542	N/A	130 (8.43%)
All with a promoter			722	53 (7.3%)	96 (13.3%)
LTR ChIPSeq+			101	23 (22.8%)	35 (34.7%)
nonLTR ChIPSeq+			109	11 (10.1%)	17 (15.6%)
LTR ChIPSeq-			185	9 (4.9%)	19 (10.3%)
nonLTR ChIPSeq-			327	10 (3.1%)	25 (7.7%)

Supplementary Figure 8

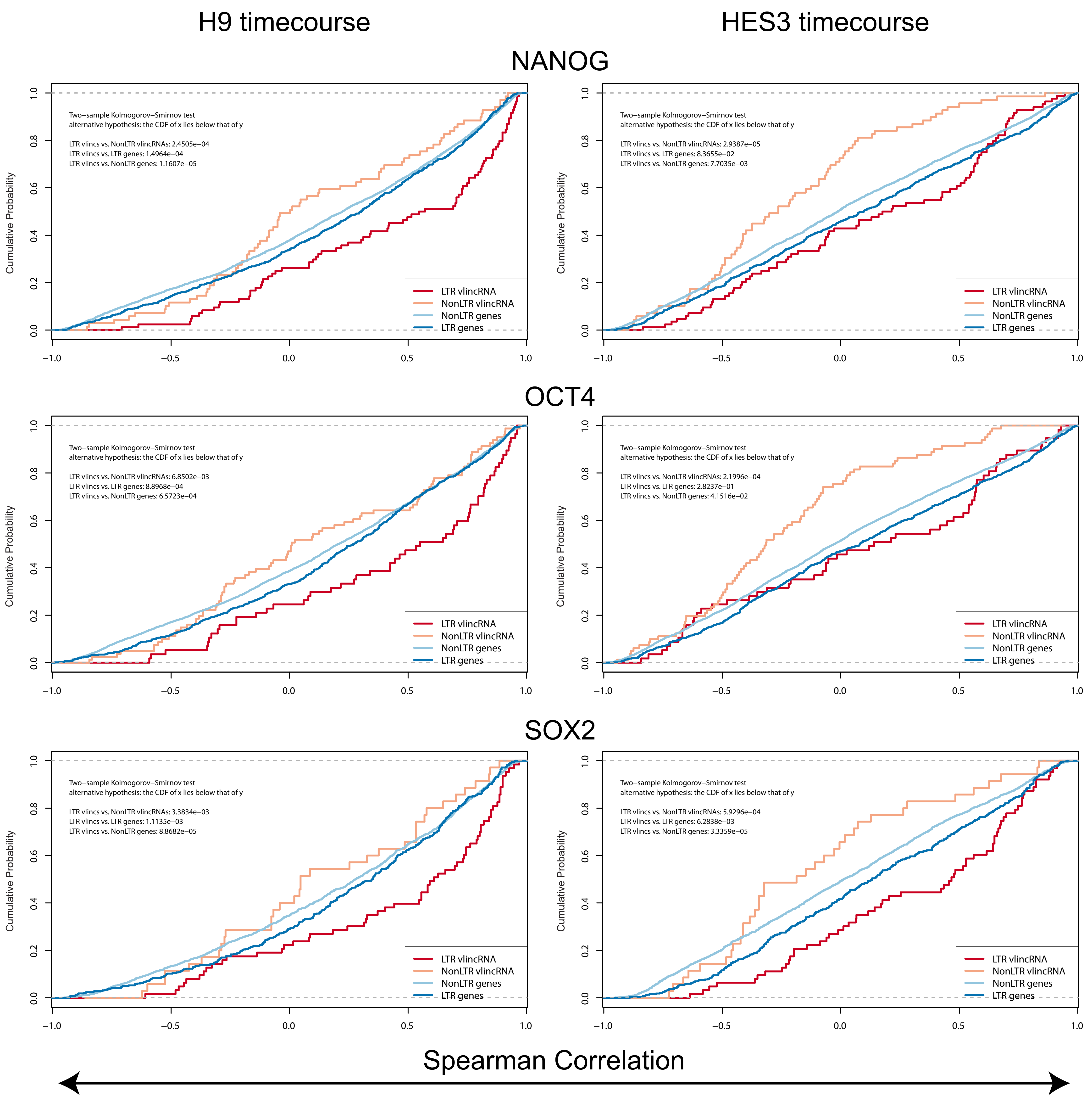

Supplementary Figure 9
